# Gut dysbiosis conveys psychological stress to activate LRP5/β-catenin pathway promoting cancer stemness

**DOI:** 10.1038/s41392-025-02159-1

**Published:** 2025-03-05

**Authors:** Bai Cui, Huandong Luo, Bin He, Xinyu Liu, Dekang Lv, Xiaoyu Zhang, Keyu Su, Sijia Zheng, Jinxin Lu, Cenxin Wang, Yuqing Yang, Zhuoran Zhao, Xianxian Liu, Xu Wang, Yingrui Zhao, Xiaoshan Nie, Yuanyuan Jiang, Ziyu Zhang, Congcong Liu, Xinyi Chen, Anqi Cai, Zhumeng Lv, Zhihang Liu, Fan An, Yunkun Zhang, Qiulong Yan, Keith W. Kelley, Guowang Xu, Lingzhi Xu, Quentin Liu, Fei Peng

**Affiliations:** 1 Institute of Cancer Stem Cell, Dalian Medical University, Dalian, China; 2 State Key Laboratory of Oncology in South China, Cancer Center, Sun Yat-sen University, Guangzhou, China; 3 Key Laboratory of Separation Sciences for Analytical Chemistry, National Chromatographic R&A Center, Dalian Institute of Chemical Physics, Chinese Academy of Sciences (CAS), Dalian, China; 4 Department of Pathology, The Second Hospital of Dalian Medical University, Dalian, China; 5 Department of Microbiology, College of Basic Medical Sciences, Dalian Medical University, Dalian, China; 6 Department of Pathology, College of Medicine and Department of Animal Sciences, College of ACES, University of Illinois at Urbana-Champaign, Urbana, IL USA; 7 Department of Oncology, the Second Affiliated Hospital, Dalian Medical University, Dalian, China

**Keywords:** Cancer metabolism, Cancer stem cells

## Abstract

Psychological stress causes gut microbial dysbiosis and cancer progression, yet how gut microbiota determines psychological stress-induced tumor development remains unclear. Here we showed that psychological stress promotes breast tumor growth and cancer stemness, an outcome that depends on gut microbiota in germ-free and antibiotic-treated mice. Metagenomic and metabolomic analyses revealed that psychological stress markedly alters the composition and abundance of gut microbiota, especially *Akkermansia muciniphila* (*A. muciniphila*), and decreases short-chain fatty acid butyrate. Supplement of active *A. muciniphila*, butyrate or a butyrate-producing high fiber diet dramatically reversed the oncogenic property and anxiety-like behavior of psychological stress in a murine spontaneous tumor model or an orthotopic tumor model. Mechanistically, RNA sequencing analysis screened out that butyrate decreases LRP5 expression to block the activation of Wnt/β-catenin signaling pathway, dampening breast cancer stemness. Moreover, butyrate as a HDAC inhibitor elevated histone H3K9 acetylation level to transcriptionally activate ZFP36, which further accelerates LRP5 mRNA decay by binding adenine uridine-rich (AU-rich) elements of LRP5 transcript. Clinically, fecal *A. muciniphila* and serum butyrate were inversely correlated with tumoral LRP5/β-catenin expression, poor prognosis and negative mood in breast cancer patients. Altogether, our findings uncover a microbiota-dependent mechanism of psychological stress-triggered cancer stemness, and provide both clinical biomarkers and potential therapeutic avenues for cancer patients undergoing psychological stress.

## Introduction

The rapidly evolving lifestyle in current society, environmental changes and income disparities cause a pervasive sense of anxiety and stress on a global scale. Psychological stress-induced anxiety or depression-like status is an increasingly recognized symptom, which is positively correlated with elevated risk and poor prognosis in cancer patients.^1^ Accumulating studies demonstrate that psychological stress stimulates the activity of central nervous system (CNS) and peripheral nervous system (PNS) to trigger dysregulation of stress-related hormones leading to cancer malignant progressions, such as proliferation, angiogenesis, metastasis and immune escape.^2^ Our recent study reveals that chronic mental stress elevates serumal and tumoral epinephrine levels to activate lactate dehydrogenase A (LDHA)-mediated glycolysis, and the resulting lactate-enriched acidic microenvironment enhances breast cancer stemness.^3^ Accumulating studies have summarized that cancer stem-like cells (CSCs), correlated with tumor aggressiveness, act as driving force for tumor initiation, cancer metastasis, drug resistance and tumor recurrence.^4–6^ Hence, the systematic regulatory mechanisms by which psychological stress induces cancer stem-like characteristics is worth further investigating.

Gut microbial dysbiosis has emerged as a consensual hallmark of cancer, attributable to its multifaceted influence on a spectrum of tumor-associated phenotypes including inflammation landscape, immune evasion microenvironment, and genomic instability that underlies the malignant transformation of tissues.^7^ A clinical study reveals that the distinct commensal from the gut microbiome impacts cancer prognosis and influences numerous side effects of cancer therapies.^8^ For instance, *Bacteroides fragilis* and *Fusobacterium nucleatum* excite the inflammatory microenvironment to trigger cancer progression by producing proinflammatory toxic factors and prevent antitumor immune functions.^9,10^ In addition, gut microbes have been shown to regulate cancer stem-like traits. A recent study unveils that *Bacterium Fusobacterium nucleatum* induces formate secretion to activate the AhR signaling pathway, leading to enrichment of cancer stem cells.^11^ In contrast, *Lactobacillus plantarum* (LP) supernatant inhibits the expression of particular CSC markers, sphere formation capacity and boosts 5-fluorouracil (5-FU) drug sensitivity in colorectal cancer cells.^12,13^ Although emerging studies verify that gut microbiota-derived secondary metabolites regulate a series of CSC properties, the molecular mechanisms of cancer stemness regulated by gut bacteria are poorly understood. Accumulating evidence suggests that chronic mental stress exhibits positive correlation with the disruptions of both physiological function and mucosal integrity in the intestine, which promotes gut bacterial dysbiosis. A recent study reveals that long-term psychological stress disrupts microbial homeostasis, accompanied by the disrupted kynurenine metabolic process and neuroendocrine homeostasis via the gut-brain circuit, to promote the development of depression-like behavior.^14^ In addition, an herbal formula, *xiao-chai-hu-tang* (XCHT) improves gut microbiota composition to attenuate cancer progression and depressive-like behavior by stimulating the activation of TLR4/MyD88/NF-κB axis, suggesting modulation of gut microbiota has a key effect on the treatment of both cancer and cancer-associated mental disorders.^15^ Therefore, the identification and characterization of special bacterial species that mediate psychological stress-induced tumor development and cancer stemness will provide novel and targeted therapeutic strategies for breast cancer patients.

Low-density lipoprotein receptor-related protein 5 (LRP5), a key member of the low-density lipoprotein receptor-related protein (LRP) family, functions as an essential co-receptor in concert with the Frizzled family of transmembrane receptors to initiate the β-catenin dependent Wnt signaling pathway.^16^ The hyperactivation of Wnt signaling pathway has emerged as a key factor that plays a critical role in the promotion and maintenance of cancer stemness across a broad spectrum of malignancies.^17,18^ LRP5 has been recently reported to promote cancer stem-like characteristics and the development of drug resistance in various cancer types.^19,20^ Moreover, it has been reported that the supplement of probiotic microbiota serves to augment the LRP5-mediated Wnt signaling pathway to facilitate the differentiation and proliferation of intestinal epithelial cells in neonatal rat models, which implies a fundamental and critical role of LRP5 in the regulation of microbiota-dependent physiological processes.^21^ Yet, the specific molecular mechanism of LRP5 in mediating gut microbiota-regulated cancer stem-like properties remains elusive.

In the current study, our findings demonstrate that tumor growth and cancer stemness caused by psychological stress is mediated by gut microbiota in germ-free and antibiotic-treated mice. Using metagenomics and metabolomic analyses, we further identify a significant reduction of *A. muciniphila* and *A. muciniphila*-related metabolite butyrate in gut microbes of mice subjected to psychological stress. Moreover, the administration of *A. muciniphila*, the provision of butyrate, and the implementation of a butyreate producing high-fiber diet, markedly reverse stress-triggered tumor growth and the acquisition of cancer stemness. In addition, we discover that butyrate, as a HDAC inhibitor, elevates histone H3K9 acetylation of the AU-rich element binding protein ZFP36 to promote its transcriptional activity. Increased ZFP36 further binding to AU-rich elements in 3’-untranslational region of LRP5 transcript to accelerate the mRNA decay of *LRP5*, thereby suppressing the activation of Wnt/β-catenin signaling-mediated enhancement of cancer stem-like traits. In the clinic, fecal *A. muciniphila* and serum butyrate are inversely correlated with tumoral LRP5/β-catenin expression and negative mood in breast cancer patients, conferring a better cancer prognosis. Hence, our study uncovers the requirement of gut microbiota in the ability of psychological stress to trigger cancer progression and cancer stem cell maintenance. *A. muciniphila* and butyrate therefore serve not only as anti-tumoral biomarkers but also provide an exciting potential therapeutic strategy for breast cancer patients suffering from negative mood disorders.

## Results

### Gut microbiota is required for psychological stress-triggered tumor development and cancer stem-like traits

**Fig. 1:**
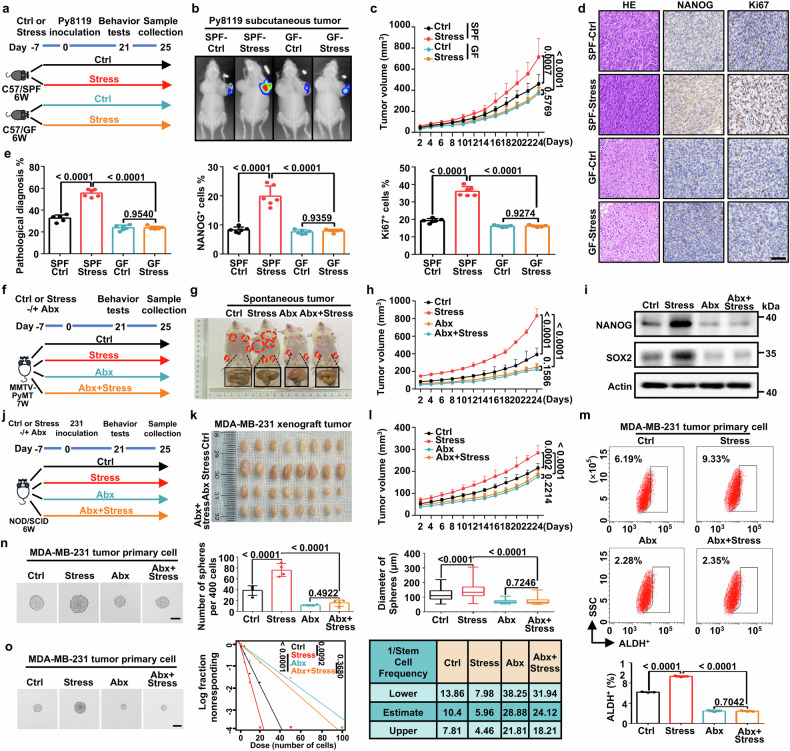
Gut microbiota is required for psychological stress-induced breast cancer progression and stemness. **a** Schematic of specific pathogen-free (SPF) and germ-free (GF) mice that were stressed (Stress) or served as control (Ctrl) (*n* = 10 mice per group). The stress group was exposed to chronic restraint stress for 7 days before inoculation with Py8119-pLVX-MCS-Luc2 or PY8119 cells, and Ctrl group was inoculated with Py8119-pLVX-MCS-Luc2 or PY8119 cells without stress exposure. **b** Representative bioluminescence images of Py8119-pLVX-MCS-Luc2 subcutaneous tumors in SPF and GF mice that were stressed or served as Ctrl. **c** Tumor volume curves in SPF/GF C57BL/6 J Ctrl or Stress mice (*n* = 10 mice per group). **d** Representative Hematoxylin-eosin (HE) photomicrographs, NANOG and Ki67 immunohistochemical (IHC) staining in Py8119-pLVX-MCS-Luc2 subcutaneous tumors (Scale bar, 50 μm). **e** Quantitative results of HE and IHC staining from indicated markers in indicated groups (*n* = 6 representative images). **f** Schematic of MMTV-PyMT mice in Ctrl and Stress groups treated with an antibiotic cocktail (Abx) or H_2_O (*n* = 6 mice per group). The Abx and Abx+Stress groups received Abx treatment throughout the experiment for 32 days. **g** Representative images of the MMTV-PyMT mice treated with Abx or H_2_O in Ctrl and Stress groups. **h** Tumor volumes curves in the Ctrl and Stress groups treated with Abx or H_2_O (*n* = 6 mice per group). **i** Relative protein levels of NANOG and SOX2 in MMTV-PyMT tumors. **j** Schematic of the NOD/SCID mice under Ctrl or Stress conditions. Mice were orthotopically injected with 1×10^6^ MDA-MB-231 cells and treated with Abx or H_2_O (*n* = 8 mice per group). The Abx and Abx+Stress groups received Abx treatment throughout the experiment for 32 days. NOD/SCID mice were orthotopically inoculated with MDA-MB-231 cells and treated with Abx or H_2_O in the Ctrl and Stress groups. Representative images of MDA-MB-231 xenograft tumors (**k**) and tumor volumes curves (**l**) are shown (*n* = 8 mice per group). **m** ALDH^+^ cells in MDA-MB-231 primary tumors (*n* = 3 biological replicates). **n** Representative images of spheroid formed by a single MDA-MB-231 tumor primary cell (left). Data represent the number of spheres per 400 cells (d > 50 μm) (middle) and the diameter of spheres (right) (Scale bars, 100 μm). **o** Extreme limiting dilution assays (ELDA) were performed in MDA-MB-231 tumor primary cells. Representative sphere images are shown (Scale bars, 50 μm). Stemness frequency of cells with the upper and lower 95% confidence intervals that shows the frequency of one stem cell in the tumors. Spheres were counted from 24 replicate wells. All data represent the mean ± s.d. (**c**, **h**, **l**) by two-tailed, unpaired Student’s *t*-test at the ethical endpoint, **e**, **m**, **n** by one-way analysis of variance (ANOVA), **o** by the likelihood ratio test. *P* values are as indicated

To investigate the potential impact of gut microbiota on psychological stress-induced tumor development, we subcutaneously transplanted syngeneic tumors in both germ-free (GF) and specific pathogen free (SPF) mice undergoing chronic restraint stress (CRS) (Fig. 1a). Chronically stressed mice exhibited anxiety-like behavior, which manifested as reduced exploration in the central area of the open-field test (OFT) and diminished exploration in the open arms of the elevated plus maze (EPM) (Supplementary Fig. 1a, b). While there were no significant changes in anxiety-like behaviors between the control (Ctrl) and CRS (Stress) group in GF mice (Supplementary Fig. 1a, b). Furthermore, no difference in body weight was observed between Stress and Ctrl groups in either SPF or GF mice throughout the 4-week period (Supplementary Fig. 1c). The bioluminescence imaging and tumor growth curves showed that tumors in GF mice were smaller compared to those in SPF mice (Fig. 1b, c). Importantly, in SPF mice, tumors from Stress mice were larger than those from Ctrl mice, but there was no difference between Ctrl and Stress groups in GF mice (Fig. 1b, c). After euthanasia, we found that GF mice displayed the typical enlargement of the cecum compared with SPF mice (Supplementary Fig. 1d), which is consistent with previous work.^22^ Tumors from the Stress group exhibited a remarkable elevated expression of stemness factors (*Nanog* and *Sox2*) compared with tumors from Ctrl in SPF mice, but this stress-induced increase did not occur in tumors from GF mice (Supplementary Fig. 1e). Hematoxylin-eosin (HE) staining and immunohistochemical (IHC) analyses also confirmed that gastrointestinal sterility reversed stress-caused an increase in the levels of proliferation marker Ki67 and the stemness marker NANOG (Fig. 1d, e). These findings establish that gut microbiota is critical for chronic mental stress-triggered tumor development and cancer stem-like properties.

We then investigated the effect of gut microbiota on psychological stress-enhanced cancer stemness in two cancer models: (a) A spontaneous breast cancer tumor model (MMTV-PyMT) and (b) An MDA-MB-231 orthotopic xenograft tumor model in which mice were treated with the antibiotic cocktail (Abx) drinking water to deplete gut commensal bacteria (Fig. 1f, j).^23^ The Firmicutes and Actinobacteria were markedly depleted after Abx administrated, confirming the anti-bacterial effectiveness of Abx (Supplementary Fig. 1f, k). Consistent with results in GF mice, Abx treatment blocked stress-induced anxiety-like behaviors without losing body weight in any of the four group mice (Supplementary Fig. 1g–i, l–n). Moreover, Abx-treated mice displayed greater volume of the cecum (Fig. 1g and Supplementary Fig. 1o). Abx-treatment also significantly inhibited tumor growth and expression of stemness factors in comparison to conventionally raised mice (Fig. 1g–i, k, l and Supplementary Fig. 1j, p–r). Primary cells from MDA-MB-231 orthotopic xenograft tumors were digested to validate cancer stem-like phenotypes. ALDH assay showed that the stress-induced increase in ALDH^+^ cells was significantly blunted by gut sterilization (Fig. 1m). Consistently, Abx treatment markedly blocked stress-enhanced sphere formation capacity as determined by sphere formation (Fig. 1n) and the extreme limiting dilution assays (Fig. 1o). Collectively, these findings suggest that gut microbiota determines psychological stress-triggered tumor development and cancer stem-like traits. Eliminating gut commensal bacteria specifically inhibits stress-promoted growth kinetics of breast tumors.

### Deficiency of *A. muciniphila* determines psychological stress-triggered cancer stemness and anxiety-like behavior

**Fig. 2:**
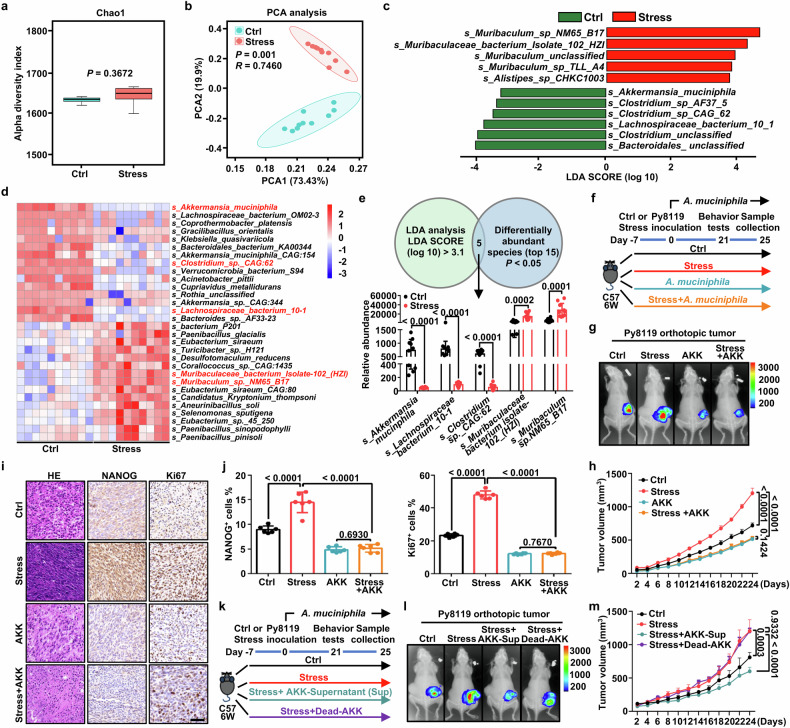
Deficiency of *A. muciniphila* determines psychological stress-triggered tumor development and anxiety-like behavior. **a** Alpha diversity of the gut microbiota between the Ctrl and Stress mice inoculated with Py8119 cells, as indicated by Chao1 indices (*n* = 10 mice per group). **b** PCA of fecal microbiota from the Ctrl and Stress mice inoculated with Py8119 cells (n = 10 mice per group). **c** Taxonomic cladogram generated from LEfSe of metagenomic sequencing data. Green indicates species level in the Ctrl mice whereas red show the species level in the Stress mice inoculated with Py8119 cells. The criteria of feature selection are log10 LDA score > 3.1. **d** Heatmap of the selected most differentially abundant features at the species level in feces of Ctrl and stressed mice inoculated with Py8119 cells (*n* = 10 mice per group). **e** Venn diagram showing the overlap of from LEfSe (log10 LDA score > 3.1) and differentially abundant species from heatmap (p < 0.05) (up). Five bacterial species abundance in Ctrl and Stress groups (down) (*n* = 10 mice per group). **f** Schematic of the Ctrl or stressed C57BL/6 J mice inoculated with Py8119 cells with *A. muciniphila* (AKK) administration. The AKK and Stress+AKK groups were treated with 200 μl (3 × 10^8^ CFU) AKK for 21 days (n = 8 mice per group). **g** Representative bioluminescence images of Py8119-pLVX-MCS-Luc2 orthotopic tumors in Ctrl and Stress groups treated with AKK or saline. **h** Tumor volumes curves in Ctrl and Stress mice treated with AKK or saline (*n* = 8 mice per group). **i** Representative HE photomicrographs, NANOG and Ki67 staining in tumors from the Ctrl and Stress group treated with AKK or saline (Scale bar, 50 μm). **j** Quantitative results of IHC staining from indicated markers in indicated groups (*n* = 6 representative images). **k** Schematic of the Ctrl or stressed C57BL/6J mice inoculated with Py8119 cells. Stressed mice were treated with 200 μl AKK-Supernatant (Sup) (3 × 10^8^ CFU) or Dead-AKK (heat-killed) for 21 days (*n* = 8 mice per group). **l** Representative bioluminescence images of Py8119-pLVX-MCS-Luc2 orthotopic tumors between Ctrl, Stress, stressed mice treated with AKK-Sup or Dead-AKK. **m** Tumor volumes curves in Ctrl and Stress mice and stressed mice treated with AKK-Sup or Dead-AKK (*n* = 8 mice per group). All data represent the mean ± s.d. (**a**, **d**, **e**, **h**, **m**) by two-tailed, unpaired Student’s *t*-test, **b** by Weighted UniFrac ANOSIM analysis, **j** by one-way ANOVA. For multiple comparisons, *p*-values were adjusted using the FDR correction. *P* values are as indicated

We further explored changes in the gut microbial landscape caused by psychological stress based on a murine subcutaneous transplanted syngeneic tumors model. Metagenomic sequencing data showed that the stool samples from SPF-Ctrl and SPF-Stress mice had no change in alpha diversity, as indicated by Chao1 (Fig. 2a). But principal component analysis (PCA) and principal coordinates analysis (PCoA) revealed that beta diversity of microbiota was substantially different between stressed mice and Ctrl mice (Fig. 2b, Supplementary Fig. 2a). Next, we analyzed structure of the microbiota community in each group and found the phylum of microbial composition dramatically changed. Intestinal Bacteroidetes was upregulated whereas Firmicutes in intestines was downregulated in stressed mice (Supplementary Fig. 2b, c). At the genus level, linear discriminant analysis effect size (LEfSe) showed a predominance of *Lachnospiraceae*, *Prevotella*, *Roseburia* and *Akkermansia* in Ctrl mice, whereas a predominance of *Muribaculum* and *Alistipes* in stressed mice. (Supplementary Fig. 2d). At the species level, LEfSe demonstrated that the Ctrl group was characterized by *A. muciniphila* and *Lachnospiraceae bacterium 10-1*, whereas the Stress group was abundant with *s_Muribaculaceae_bacterium_Isolate_102_HZI* and *Muribaculum sp. NM65_B17* (Fig. 2c). We further assessed differential abundance of bacterial species between the Ctrl and Stress group (Fig. 2d). We next overlap the LEfSe data and significant differentially abundant species, the abundance of *A. muciniphila* and *A. muciniphila* co-occurring bacterial species including *Clostridium sp._CAG:62*, *Lachnospiraceae bacterium 10-1*^24,25^ was significantly reduced in Stress mice (Fig. 2e). Meanwhile, the abundance of *Muribaculaceae bacterium isolate-102_(HZI)* and *Muribaculum sp. NM65 _B17* were significantly elevated in feces of stressed mice, which was consistent with previous study in mice without tumors (Fig. 2e).^26^ We further confirmed that *A. muciniphila* in fecal samples from Stress mice was significantly depleted compared with Ctrl mice in three different murine breast tumor models using qPCR (Supplementary Fig. 2e). By using these murine breast tumor models, the data collectively established that psychological stress dramatically changed gut microbes, predominately resulting in a reduction of *A. muciniphila*.

To determine the effect of *A. muciniphila* on ameliorating stress-induced tumor progression, we addressed intragastrically supplement of *A. muciniphila* in an orthotopic syngeneic tumor mice under CRS (Fig. 2f). Successful colonization with *A. muciniphila* was confirmed by detecting *A. muciniphila* (AKK) levels in stools using qPCR (Supplementary Fig. 3a). Behavioral assays using OFT and EPM showed that *A. muciniphila* treatment blocked CRS-induced anxiety-like behavior (Supplementary Fig. 3b, c). Body weight had no change between the mice administered with *A. muciniphila* and vehicle (Supplementary Fig. 3d). Notably, oral gavage with *A. muciniphila* also significantly prevented the increase in tumor proliferation caused by psychological stress, as evidenced by bioluminescence imaging, tumor images and tumor growth curves in both C57BL/6 J and BALB/c mice orthotopic syngeneic tumor models (Fig. 2g, h and Supplementary Fig. 3e-j). Consistently, intragastrically supplemented with another strain of *A. muciniphila* #2 (AKK-2) also blocked psychological stress-induced anxiety-like behaviors and tumor growth without affecting body weight (Supplementary Fig. 3k-p). While, randomly supplemented with the other psychological stress-related strain, *Alistipes shahii* (*A. shahii*), as a negative control exhibits no effect on anxiety-like behavior and tumor progression (Supplementary Fig. 3k-p). In orthotopic tumors, *A. muciniphila*-supplementation reversed the increase in Ki67 and stemness factors including Nanog and Sox2 caused by stress (Fig. 2i, j and Supplementary Fig. 3q). In addition, administration of *A. muciniphila* blocked the stress-enhanced sphere formation, as assessed by primary tumor cell sphere formation and extreme limiting dilution assays (Supplementary Fig. 3r, s).

We next sought to determine the mechanistic basis of how *A. muciniphila* ameliorates stress-induced tumor progression. Considering bioactive metabolites from bacteria are recognized as the major modulators of host pathophysiologic processes, we investigated whether bioactive metabolites or physical components of *A. muciniphila* are sufficient to mitigate Py8119 breast tumor outgrowth (Fig. 2k). In contrast to mice treated with the Dead-AKK by heat-killed, oral administration of the AKK-supernatant (Sup) was sufficient to inhibit psychological stress-triggered anxiety symptom and exhibited less impact on body weight in any of four group mice (Supplementary Fig. 3t-v). Also compared with the Dead-AKK treated group, mice that were supplied with the *A. muciniphila* supernatant displayed significant tumor regression (Fig. 2l, m), suggesting that *A. muciniphila* may influence antitumor effect through its metabolites.

### Administration of *A. muciniphila*-associated butyric acid reverses stress-mediated cancer stemness and anxiety-like behavior

**Fig. 3:**
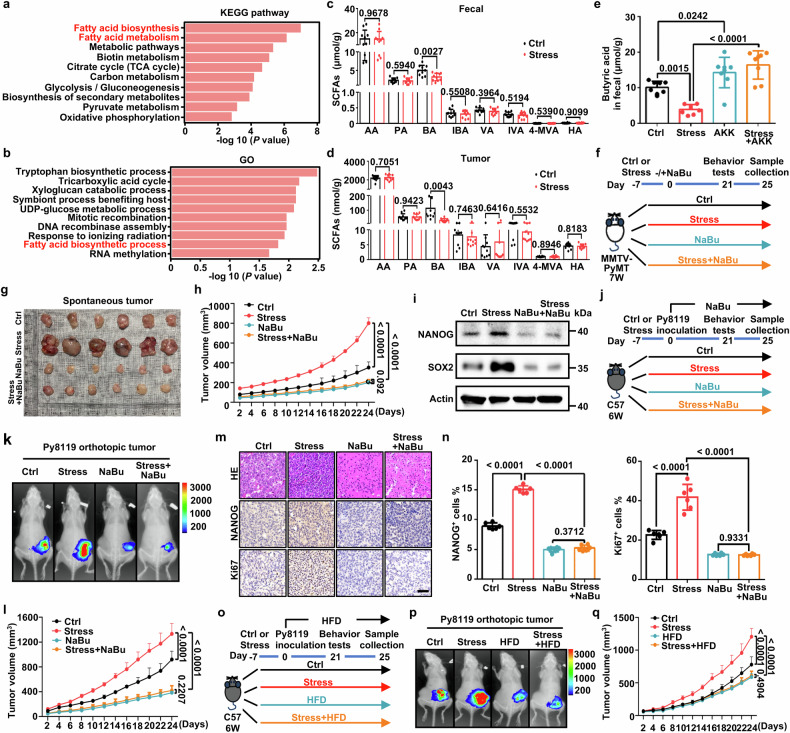
Administration of *A. muciniphila*-associated butyric acid reverses stress-mediated cancer stemness and anxiety-like behavior. **a** The enriched KEGG metabolic pathways of upregulated fecal microbiota genes in C57BL/6J mice inoculated with Py8119 tumors following stress treatment. **b** The enriched GO metabolic pathways of upregulated fecal microbiota genes in C57BL/6J mice inoculated with Py8119 tumors following stress treatment. **c** Levels of fecal short chain fatty acids (SCFAs) such as acetic acid (AA), propionic acid (PA), butyric acid (BA), isobutyric acid (IBA), valeric acid (VA), isovaleric acid (IVA), 4-methylvaleric acid (4-MVA), hexanoic acid (HA) in mice inoculated with Py8119 tumors undergoing stress paradigm (*n* = 10 mice per group). **d** Levels of multiple SCFAs in Py8119 tumors following stress treatment (*n* = 10 mice per group). **e** Levels of fecal butyric acid in mice inoculated with Py8119 tumors undergoing stress paradigm and AKK treatment (*n* = 8 mice per group). **f** Schematic of the MMTV-PyMT mice in Ctrl and Stress groups treated with 200 mg/kg sodium butyrate (NaBu) or saline (*n* = 6 mice per group). **g** Representative tumor images from MMTV-PyMT mice treated with NaBu or saline in the Ctrl and Stress groups (*n* = 6 mice per group). **h** Tumor growth curves of MMTV-PyMT mice (*n* = 6 mice per group). **i** Relative protein levels of NANOG and SOX2 in MMTV-PyMT tumors. **j** Schematic of the Ctrl or stressed C57BL/6J mice inoculated with Py8119 cells. The NaBu and NaBu+Stress groups treated with 200 mg/kg NaBu or saline (*n* = 8 mice per group) for 21 days. **k** Representative bioluminescence images of Py8119-pLVX-MCS-Luc2 orthotopic tumors in the Ctrl and Stress groups treated with NaBu or saline. **l** Tumor volumes from Ctrl and Stress mice treated with NaBu or saline (*n* = 8 mice per group). **m** Representative HE photomicrographs and NANOG and Ki67 staining from tumors of the Ctrl and Stress group treated with NaBu or saline (Scale bar, 50 μm). **n** Quantitative results IHC staining from indicated markers in indicated groups (*n* = 6 representative images). **o** Schematic of C57BL/6J mice inoculated with Py8119 cells in the Ctrl and Stress groups treated with high fiber diet (HFD) or normal diet (ND) for 21 days (*n* = 8 mice per group). **p** Representative bioluminescence images of Py8119-pLVX-MCS-Luc2 orthotopic tumors from Ctrl and Stress mice given the HFD or ND. **q** Tumor volumes from Ctrl and Stress mice treated with or without HFD (*n* = 8 mice per group). All data represent the mean ± s.d. (**a**, **b**) by Mann Whitney U test, (**c**, **d**, **h**, **l**, **q**) by two-tailed, unpaired Student’s *t*-test. **e**, **n** by one-way ANOVA. For multiple comparisons, p-values were adjusted using the FDR correction. *P* values are as indicated

We next identified the changes in microbial community function caused by stress. KEGG analysis revealed that changed microbiota genes clustered in many metabolism-related signaling pathways: fatty acid biosynthesis ranked first, followed by fatty acid metabolism and other pathways (Fig. 3a). Gene Ontology (GO) data also predicted fatty acid biosynthesis as one of the key pathways leading to gut dysbiosis caused by psychological stress (Fig. 3b). As *A. muciniphila* is associated with the production of short chain fatty acids (SCFAs),^27^ we next determined the SCFA profiles in fecal, tumor and serum samples from the murine orthotopic syngeneic tumor model. Compared to other SCFAs, psychological stress dramatically decreased levels of butyric acid in fecal, tumor and serum samples (Fig. 3c, d and Supplementary Fig. 4a). Meanwhile, intragastrically supplemented with *A. muciniphila* significantly increased the levels of butyric acid in mice fecal both in AKK and stress plus AKK groups (Fig. 3e). Further analyses between the gut microbiota and SCFAs confirmed that *A. muciniphila* and *A. muciniphila* co-abundant microorganisms were positively associated with butyric acid levels in fecal, tumors and serum samples. However, *Muribaculaceae bacterium isolate-102_(HZI)* and *Muribaculum sp. NM65 _B17* displayed a negative correlation with butyric acid levels (Supplementary Fig. 4b–d).

To further explore whether *A. muciniphila* directly produces butyric acid, we performed a targeted SCFAs metabolomics analysis of the *A. muciniphila* culture supernatant. We observed that *A. muciniphila* generated and secreted a variety of acetate, and propionate, but not butyrate (Supplementary Fig. 4e). Strikingly, intragastric treatment with *A. muciniphila* to Abx-pretreated mice had no effect on suppressing tumor progression compared with Ctrl group (Supplementary Fig. 4f–h). Meanwhile, metabolomics data showed no difference in butyric acid concentration between the Abx-AKK and Abx-Ctrl groups in mice feces (Supplementary Fig. 4i). Since our data shows a strong correlation between *A. muciniphila* and butyrate, we speculate that *A. muciniphila* creates an environment that favors the survival of butyrate-producing bacteria. Using metagenomics sequencing, we confirmed that the abundance of *A. muciniphila* accompanied with butyrate-producing bacterial species including *Anaerostipes hadrus*, *Clostridium butyricum* and *Roseburia inulinivorans*^28^ significantly increased in the AKK and stress plus AKK groups compared with control or stress groups (Supplementary Fig. 4j).

We further validate the dose-dependent function of butyrate in tumor growth. Increasing doses of butyrate were orally gavaged in Py8119 orthotopic injected mice (Supplementary Fig. 5a).^29^ The results indicated that oral NaBu dose-dependent suppressed tumor growth (Supplementary Fig. 5b, c). Furthermore, PyMT mice were intragastrically administered with vehicle or sodium butyrate (NaBu) under CRS (Fig. 3f). We found that NaBu treatment reversed CRS-enhanced tumor growth without affecting body weight (Fig. 3g, h and Supplementary Fig. 5d). The stress-induced increase in tumoral NANOG and SOX2 expression was rescued by NaBu administration (Fig. 3i and Supplementary Fig. 5e). Consistent with these results in the PyMT tumor model, NaBu treatment also blocked stress-induced anxiety-like behaviors without affecting body weight in an orthotopic tumor model (Fig. 3j and Supplementary Fig. 5f–h). We also observed that NaBu supplementation-suppressed tumor growth was similar to that of *A. muciniphila* administration in an orthotopic syngeneic tumor model (Fig. 3k, l). HE staining and IHC analysis confirmed that NaBu treatment significantly restricted the ability of psychological stress to induce high levels of the proliferation gene Ki67 and stemness genes (Nanog and Sox2) (Fig. 3m, n and Supplementary Fig. 5i). NaBu-treated tumors also reversed stress-enhanced mammosphere-forming capacity in primary cells (Supplementary Fig. 5j, k).

An earlier report showed that dietary fiber can be fermented by microbiota to produce SCFAs.^30^ This led us to investigate the possibility that a high-fiber diet (HFD) could alleviate psychological stress-induced tumor growth (Fig. 3o). As expected, administration of a HFD blocked stress-induced anxiety-like behaviors without body weight change among four groups (Supplementary Fig. 5 l–n). Moreover, HFD reversed stress-induced tumor proliferation as evidenced by live imaging and tumor growth curves (Fig. 3p, q). Using metagenomics sequencing, we verified that both *A. muciniphila* and butyrate-producing bacteria including *Clostridium acetobutylicum*, *Anaerostipes hadrus, Eubacterium hallii*, and *Faecalibacterium prausnitzii*^28^ were significantly increased in HFD or stress plus HFD groups (Supplementary Fig. 5o). The targeted fecal metabolites SCFAs analysis showed that HFD treatment significantly upregulated the butyrate levels in the murine fecal samples (Supplementary Fig. 5p). In addition, HFD reversed stress-upregulated stemness-related gene expression (Supplementary Fig. 5q). Collectively, our results demonstrate that administration of *A. muciniphila*, NaBu or a HFD significantly restrains stress-induced tumorigenesis, anxiety-like behaviors and stem-like properties.

### Blockage of LRP5-mediated Wnt/β-catenin signaling via butyrate inhibits cancer stemness

**Fig. 4:**
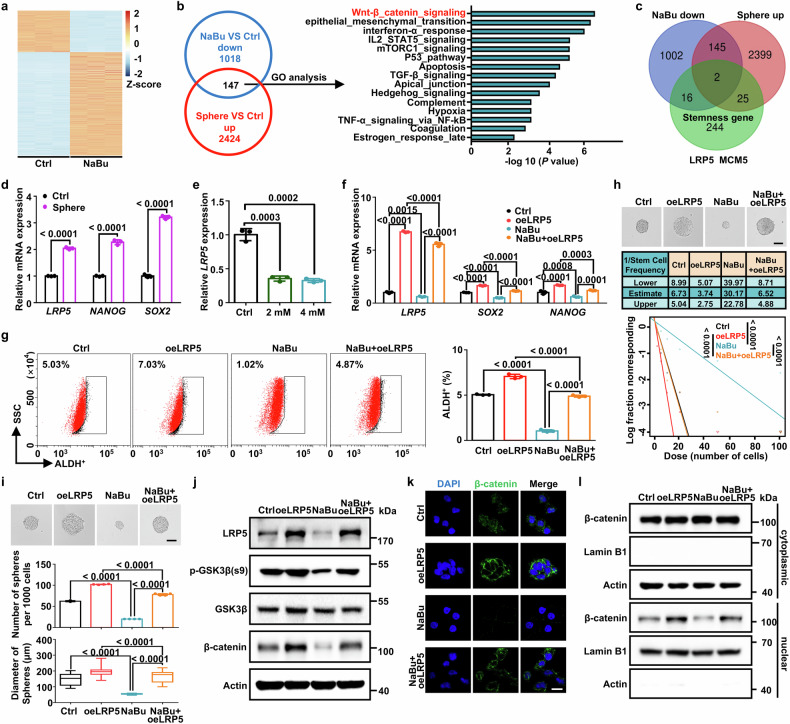
Blockage of LRP5-mediated Wnt/β-catenin signaling via butyrate inhibits cancer stemness. **a** Heatmap showing differentially expressed genes (DEGs) between untreated SK-BR-3 cells and SK-BR-3 cells treated with 4 mM NaBu for 48 hours. The number of significant variant genes (FC > 2) are shown (FC, fold change) (*n* = 3 biological replicates). **b** Venn diagram showing the overlap of downregulated genes from NaBu-treated SK-BR-3 cells (FC > 2, *P* < 0.05, 1165 genes) and upregulated genes in MDA-MB-231 spheres (FC > 2, *P* < 0.05, 2571 genes) and the subsequent GO analysis. **c** Venn diagram showing the overlap of downregulated genes from NaBu-treated SK-BR-3 cells, upregulated genes in MDA-MB-231 spheres and stemness genes. **d** Expression of mRNA for the indicated genes in SK-BR-3 Ctrl cells and spheres (*n* = 3 biological replicates). **e** Relative *LRP5* mRNA expression in SK-BR-3 cells treated with NaBu for 48 h in dose-dependent manner (*n* = 3 biological replicates). **f** Relative mRNA expression of *LRP5*, *SOX2*, and *NANOG* in Ctrl and LRP5-forced expression SK-BR-3 cells treated with NaBu (4 mM) for 48 h or vehicle (*n* = 3 biological replicates). **g** ALDH^+^ cells in Ctrl and LRP5-forced expression SK-BR-3 cells treated with NaBu (4 mM) for 48 h or vehicle (*n* = 3 biological replicates). **h** ELDA was performed in Ctrl and LRP5-forced expression SK-BR-3 cells treated with NaBu (4 mM) for 48 h or vehicle, and representative sphere images are shown (Scale bars, 50 μm). Stemness frequency with the upper and lower 95% confidence intervals that indicate the frequency of one stem cell in tumors. Spheres were counted from 24 replicate wells. **i** Representative images of spheroids formed by single cells in the 4 groups (Scale bars, 100 μm) (up), number of spheres per 1000 cells (d > 50 μm) (middle), and distribution pattern of sphere diameter (down). **j** Relative LRP5, p-GSK3β, GSK3β and β-catenin protein levels in Ctrl and LRP5-forced expression SK-BR-3 cells treated with NaBu (4 mM) for 48 h or vehicle. **k** Representative immunofluorescence images of β-catenin protein (green) in the 4 groups, with nuclear staining with DAPI (blue) (Scale bars, 25 μm). **l** Western blot detects the expression of nuclear and cytoplasmic protein extracts from Ctrl and LRP5 forced expression SK-BR-3 cells treated with NaBu (4 mM) for 48 h or vehicle. Actin was used as a cytoplasmic internal loading control and Lamin B1 as the nuclear internal control. Results are presented as mean ± s.d. (**a,** **d,** **e**) by a two-tailed, unpaired Student’s *t-*test, (**f**, **g**, **i**) by one-way ANOVA, **h** by the likelihood ratio test. For multiple comparisons, p-values were adjusted using the FDR correction. *P* values are as indicated

To explore the potential mechanism of NaBu-inhibited stem-like traits, we conducted RNA sequencing (RNA-seq) on SK-BR-3 cancer cells treated with NaBu. Distinct transcriptomic profiles were observed between the two groups of cells (Fig. 4a). We next overlapped the downregulated genes from NaBu-treated RNA-seq results with the upregulated genes from the spheroid-enriched cancer stem-like cells (CSCs) RNA-seq dataset (GSE116180). Through Gene Ontology (GO) analysis, the overlapped 147 genes were considered to be involved functionally in the signaling pathway of Wnt/beta-catenin (Fig. 4b). To explore a key target that responds to NaBu, we further overlapped 147 genes with the stemness geneset^31^ and identified two oncogenes, *LRP5* and *MCM5* (Fig. 4c). LRP5 and Frizzled (FZD) family are canonical co-receptors that stimulates the activity of Wnt/β-catenin,^32^ which generally facilitates carcinogenesis and stemness maintenance.^33,34^ Indeed, we comfirmed that *LRP5* mRNA expression was significantly enriched in spheres cells (Fig. 4d and Supplementary Fig. 6a). CCK8 assay showed that NaBu treatment (2 mM or 4 mM) had little effect on cell viability of breast cancer (Supplementary Fig. 6b), while dose-dependently inhibited *LRP5* mRNA level (Fig. 4e and Supplementary Fig. 6c). In murine tumor samples, we validated that CRS also promotes expression of LRP5 and β-catenin in tumors, and this effect is reversed by gut sterilization (Supplementary Fig. 6d–i). Importantly, the reduction in the stemness factors *SOX2* and *NANOG* caused by NaBu was reversed by overexpression of *LRP5* but not *MCM5* (Fig. 4f and Supplementary Fig. 6j, k). Meanwhile, forced expression of *LRP5* but not *MCM5* rescued NaBu-decreased ALDH^+^ cells (Fig. 4g and Supplementary Fig. 6l, m). Additionally, aberrant expression of *LRP5* significantly rescued NaBu-inhibited capacity of sphere formation (Fig. 4h, i and Supplementary Fig. 6n, o). Finally, phosphorylation of the LRP5 adaptor protein GSK3β at serine 9 (inactive form) has recently been shown to be critical for β-catenin stability and subsequent nuclear localization to activate target gene transcription.^33,35^ Indeed, we showed that *LRP5* overexpression abrogated NaBu-inhibited GSK3β phosphorylation (Ser 9) to restore β-catenin levels (Fig. 4j and Supplementary Fig. 6p). Forced expression of *LRP5* also restored nuclear localization of β-catenin that was suppressed by NaBu treatment. (Fig. 4k, l and Supplementary Fig. 6q, r). Taken together, butyrate suppresses LRP5 expression to block Wnt/β-catenin signaling activity, reducing breast cancer stemness.

### Butyrate accelerates LRP5 mRNA decay through transactivating ARE binding protein ZFP36

**Fig. 5:**
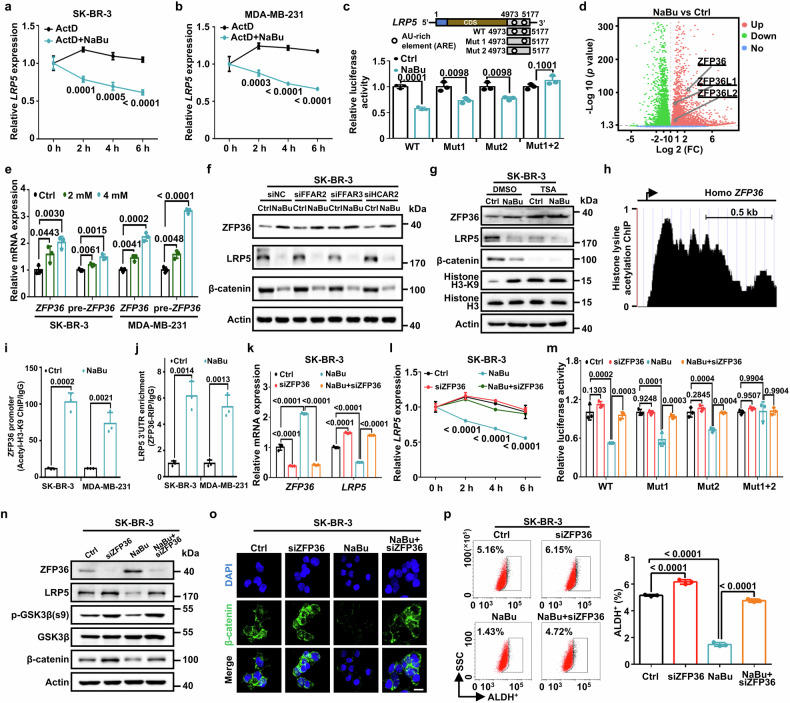
Butyrate accelerates LRP5 mRNA decay by transactivating ARE binding protein ZFP36. **a** Stability of *LRP5* mRNA in SK-BR-3 cells treated with NaBu (4 mM) or vehicle (*n* = 3 biological replicates). **b** Stability of *LRP5* mRNA in MDA-MB-231 cells treated with 4 mM NaBu or vehicle (*n* = 3 biological replicates). **c** Relative luciferase activity of psiCHECK2-LRP5-3’UTR-WT, Mut1, Mut2, and Mut1 + 2 in HEK293T cells treated with NaBu (4 mM) or vehicle (*n* = 3 biological replicates). **d** Volcano plots displaying DEGs comparing SK-BR-3 cells treated with NaBu (4 mM) or vehicle. The number of significant variant genes (FC > 2, *P* < 0.05) are shown. **e** Relative *ZFP36* and pre-*ZFP36* mRNA levels of SK-BR-3 and MDA-MB-231 cells treated with NaBu for 48 h in dose-dependent manner (*n* = 3 biological replicates). **f** Relative ZFP36, LRP5, and β-catenin protein levels in SK-BR-3 cells after transfection with the siRNA targeting these three GPRs and treated with NaBu (4 mM). **g** Relative ZFP36, LRP5, β-catenin, Histone H3-K9, and Histone H3 protein levels in SK-BR-3 cells treated with NaBu (4 mM) and TSA (1 μM; MCE, HY-15144). **h** Genome browser images showing ChIP-seq signals for the ZFP36 promoter using data from the Cistrome Data Browse (GSM810671). **i** Relative fold change of the ZFP36 promoter binding motif in SK-BR-3 and MDA-MB-231 cells treated with NaBu (4 mM) for 48 h or vehicle as analyzed by ChIP-qPCR (*n* = 3 biological replicates). **j** Enrichment of endogenous LRP5 3’UTR in SK-BR-3 and MDA-MB-231 cells treated with NaBu (4 mM) for 48 h or vehicle was analyzed by RNA immunoprecipitation (RIP) (*n* = 3 biological replicates). Relative *LRP5* and *ZFP36* mRNA expression (**k**) and stability of *LRP5* mRNA (**l**) in SK-BR-3 cells treated with knockdown of ZFP36 and NaBu (4 mM), as assessed by RT-qPCR (*n* = 3 biological replicates). **m** Relative luciferase activity of psiCHECK2-LRP5-3’UTR-WT, Mut1, Mut2, and Mut1 + 2 in HEK293T cells treated with ZFP36 knocked down and NaBu (4 mM) (*n* = 3 biological replicates). **n** Relative ZFP36, LRP5, p-GSK3β, GSK3β, and β-catenin protein levels in SK-BR-3 cells treated with ZFP36 knocked down and NaBu (4 mM). **o** Representative immunofluorescence images of β-catenin protein (green) in SK-BR-3 cells treated with ZFP36 knocked down and NaBu (4 mM). The nucleus was stained with DAPI (blue) (Scale bars, 25 μm). **p** ALDH^+^ SK-BR-3 cells treated with ZFP36 knocked down and NaBu (4 mM) (*n* = 3 biological replicates). Results are presented as mean ± s.d. (**a**–**e,** **i**, **j**) by a two-tailed, unpaired Student’s *t-*test, (**k**, **l**, **m**, **p**) by one-way ANOVA. For multiple comparisons, p-values were adjusted using the FDR correction. *P* values are as indicated

To explore the mechanism by which NaBu decreases *LRP5* expression, we first tested the possibility that NaBu inhibits LRP5 transcription. The level of *LRP5* pre-mRNA in NaBu-treated cells remained unchanged compared with no-treated cells (Supplementary Fig. 7a, b), indicating that NaBu had no effect on *LRP5* transcription. We then verified that NaBu accelerated *LRP5* mRNA decay (Fig. 5a, b). As AU-rich elements (ARE) containing AUUUA motifs generally in mRNA 3’-untranslated regions are recognized by ARE binding proteins to determine mRNA decay,^36^ we identified two AREs in the 3’UTR of *LRP5* mature mRNA (Fig. 5c, top). We next constructed and tested these serial luciferase reporters with the *LRP5* 3’UTR (WT, Mut1, Mut2, Mut1 + 2). These experiments showed that NaBu reduced luciferase activity of the WT, Mut1 and Mut2 constructs but not that of the Mut1 + 2 reporter (Fig. 5c, bottom). These results indicate that the AREs in 3’ UTR is responsible for butyrate promoting *LRP5* mRNA decay.

To further investigate the specific ARE binding protein mediating NaBu-induced LRP5 mRNA degradation, we analyzed the NaBu-treated RNA-seq data in order to identify canonical ARE binding proteins. These included ZFP36/TTP (ZFP36 ring finger protein), ZFP36L1/BRF1 and ZFP36L2/BRF2,^36^ all of which were dramatically enriched following NaBu treatment (Fig. 5d). To validate these results, we then validated mRNA levels of *ZFP36*, *ZFP36L1* and *ZFP36L2* in NaBu-treated cells. We found that mRNA levels of *ZFP36* and pre-*ZFP36*, but not *ZFP36L1* and *ZFP36L2*, were significantly upregulated by NaBu (Fig. 5e and Supplementary Fig. 7c–e). Consistently, protein expression of ZFP36 was also upregulated by NaBu (Supplementary Fig. 7f). Mechanistically, butyrate generally activates G protein coupled receptors (GPRs) or block the activity of histone deacetylases (HDACs).^37^ Ablation of 3 butyrate-related GPRs could not vary the butyrate-upregulated ZFP36 level and butyrate-blocked LRP5/β-catenin signaling (Fig. 5f and Supplementary Fig. 7g), suggesting that butyrate may not boost ZFP36 expression through the GPR receptors. In addition, we found that the prototypical pan-HDAC inhibitor trichostatin A (TSA) markedly blocked the effect of butyrate in increased acetylation at histone H3-K9 site, upregulation of ZFP36 levels and downregulation of LRP5/β-catenin signaling (Fig. 5g and Supplementary Fig. 7h). Together, our findings show that butyrate promotes ZFP36 transcription via HDAC inhibitory activity. We further confirmed that significant histone acetylation peaks surrounding the *ZFP36* promoter were observed in the ChIP-seq data^38^ from the Cistrome Data Browser (Fig. 5h).^39^ Acetyl-H3-K9 ChIP assay further confirmed that NaBu elevated acetylation of histone H3K9 (H3K9ac) located in ZFP36 promoter (Fig. 5i). We also found that ZFP36 binds to the ARE region of the endogenous LRP5 3’UTR as assessed using the RIP assay (Fig. 5j). Moreover, depletion of ZFP36 significantly increased *LRP5* mRNA and rescued the NaBu-induced reduction in LRP5 (Fig. 5k and Supplementary Fig. 7i). Ablation of *ZFP36* rescued NaBu-induced *LRP5* mRNA degradation (Fig. 5l and Supplementary Fig. 7j). Luciferase reporter assays also showed that silencing of *ZFP36* restored the NaBu-attenuated LRP5 3’UTR luciferase activity (Fig. 5m). Furthermore, knockdown of ZFP36 increased protein expression of phosphorylated GSK3β at S9, LRP5 and β-catenin in NaBu-treated cells, suggesting that ZFP36 regulates the NaBu-inhibited LRP5/β-catenin pathway (Fig. 5n and Supplementary Fig. 7k). Indeed, depletion of *ZFP36* rescued the NaBu-suppressed β-catenin nuclear localization (Fig. 5o and Supplementary Fig. 7l). Furthermore, *ZFP36* deficiency markedly rescued the ability of NaBu to reduce expression of stemness factors NANOG and SOX2 (Supplementary Fig. 7m, n) as well as the NaBu-inhibited ALDH^+^ cell population (Fig. 5p and Supplementary Fig. 7o). Collectively, these results demonstrate that the elevation in ZFP36 caused by butyrate destabilizes LRP5 mRNA to suppress β-catenin activation, thereby suppressing cancer stem-like traits.

### Clinical relevance of *A. muciniphila*, butyric acid and LRP5/β-catenin expression in breast cancer patients with negative mood

Encouraged by the above findings, we next evaluated the clinical relevance of psychological states, *A. muciniphila* abundance, butyric acid levels and LRP5/β-catenin expression in cohort 1 breast cancer patients. We recruited 57 hospital inpatients and assessed anxiety and depression symptoms using the self-administered Hospital Anxiety and Depression Scale (HADS).^40,41^ Based on the score of subscales (0-42 score), we divided the patients into two groups: HADS low (score ≤ 7, no anxiety or depression symptoms) and HADS high (score > 7, present with anxiety or depression symptoms).^42^ The relationship between HADS score and clinical-pathological parameters was then analyzed (Supplementary Table 1). Patients with high HADS scores were significantly correlated with tumor node status, distant metastasis, stage, specific biomarker of breast cancer CA153 and serum stress-related hormones including norepinephrine, estradiol, cortisol and thyroid hormone (Supplementary Table 2).

**Fig. 6:**
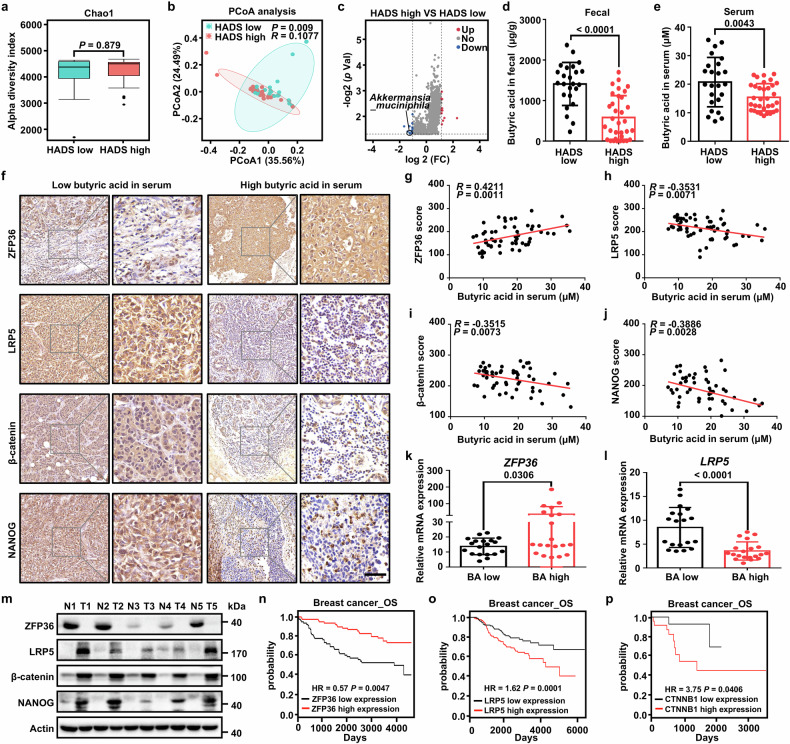
Clinical relevance of *A. muciniphila*, butyric acid and LRP5/β-catenin expression in breast cancer patients with negative mood. **a** Alpha diversity of gut microbiota between the HADS low (*n* = 24) and high (*n* = 33) groups of cohort 1 breast cancer patients, as indicated by Chao1 indices. **b** PCoA of fecal microbiota from cohort 1 breast cancer patients in the HADS low (*n* = 24) and high (*n* = 33) groups. **c** Volcano plots of the species level in the patients’ feces between the HADS low (*n* = 24) and high (*n* = 33) groups. The number of significant variant genes (*P* < 0.05) are shown. Levels of fecal butyric acid (**d**) and serum butyric acid (**e**) in cohort 1 breast cancer patients in the HADS low (*n* = 24) and HADS high (*n* = 33) groups. **f** Representative ZFP36, LRP5, β-catenin, and NANOG IHC staining images of tumors in patients in the low and high serum butyric acid groups (Scale bar, 50 μm). Pearson correlation between butyric acid concentrations of feces and ZFP36 (**g**), LRP5 (**h**), β-catenin (**i**) and NANOG (**j**) scores in breast cancer patients (*n* = 57). Statistical significance was determined by Pearson’s rank test. Relative *ZFP36* (**k**) and *LRP5* (**l**) mRNA expression between butyric acid in serum low (*n* = 19) and high (*n* = 20) groups in cohort 1 breast cancer patients. **m** Immunoblot analysis of proteins in breast tumor tissues (T) and adjacent normal breast tissues (N) (*n* = 5). Kaplan-Meier estimates of overall survival (OS) based on the expression of *ZFP36* (**n**), *LRP5* (**o**) and *CTNNB1* (**p**). Patients were stratified into high-expression and low-expression groups using the median gene expression value as the cutoff in breast cancer patients. Results are presented as mean ± s.d. (**a**, **c**, **d**, **e**, **k**, **l**) by a two-tailed, unpaired Student’s *t-*test, (**b**) by weighted UniFrac ANOSIM analysis, **g**–**j** by Pearson’s rank test. For multiple comparisons, p-values were adjusted using the FDR correction. *P* values are as indicated

We further explored alterations of gut microbes in cohort 1 breast cancer patients with anxiety/depression-like characteristics, we performed shotgun metagenomics sequencing using stool samples from breast cancer patients. Bacterial alpha diversity did not change between HADS high and HADS low patients (Fig. 6a). While, beta diversity exhibited significant difference between HADS high and HADS low patients (Fig. 6b). Further evaluation of abundance using volcano plots verified the significant decrease of *A. muciniphila* in the HADS high patients (Fig. 6c). Correlation analysis of HADS score and *A. muciniphila* level revealed that HADS was negatively correlated with *A. muciniphila* (Supplementary Fig. 8a). Among the cohort 1 clinical-pathological parameters, we divided 57 breast cancer samples into two groups, including *A. muciniphila* low (abundance ≤ 23.54) and *A. muciniphila* high (abundance > 23.54), the cutoff based on the median of *A. muciniphila* abundance in patient fecal metagenomics data.^43^ *A. muciniphila* high was negatively correlated with tumor distant metastasis, stage and the specific biomarker of breast cancer CA153 (Table 1).

**Table 1:** Correlation between *Akkermansia muciniphila* and clinicopathological parameters

Factor	*A. muciniphila* high		*A. muciniphila* low		*P* value
	NO.	%	NO.	%	
All patients	28	49.1	29	50.9	
**Age**, years					1
≤54	15	50	15	50	
>54	13	48.1	14	51.9	
**Tumor size**, cm					0.9
≤1	15	51.7	14	48.3	
>1	13	46.4	15	53.6	
**Node status**					1
Negative	9	50	9	50	
Positive	19	48.7	20	51.3	
**Distant metastasis**					0.005
Negative	22	66.7	11	33.3	
Positive	6	25	18	75	
**Stage**					0.005
I or II	22	66.7	11	33.3	
III	6	25	18	75	
**ER status**					1
Negative	7	70	3	30	
Positive	21	44.7	26	55.3	
**PR status**					0.4
Negative	7	63.6	4	36.4	
Positive	20	44.4	25	55.6	
Missing	1				
**HER2 status**					1
Negative	3	42.9	4	57.1	
Positive	23	47.9	25	52.1	
Missing	2				
**Ki67 status**					1
≤0.3	14	50	14	50	
>0.3	10	50	10	50	
Missing	4		5		
**CA153**, U/mL					<0.0001
≤9.48	22	75.9	7	24.1	
>9.48	6	21.4	22	78.6	

Statistical significance was determined by Pearson’s Chi-squared (*χ*^*2*^) test and Fisher’s Exact Test (ER status and HER2 status)

The level of fecal and serum SCFAs in cohort 1 breast cancer patients, especially butyric acid, was measured using GC-MS. Consistent with the findings described between HADS scores and abundance levels of *A. muciniphila*, patients with a high HADS score had lower levels of fecal and serum butyric acid (Fig. 6d, e). Notably, butyric acid levels in both fecal and serum were negatively associated with HADS score in breast cancer patients (Supplementary Fig. 8b, c). A significant association was also discovered between *A. muciniphila* abundance and butyric acid levels in patients fecal and serum with breast cancer (Supplementary Fig. 8d, e). Furthermore, low abundance of both fecal *A. muciniphila* and fecal butyric acid in HADS high groups were confirmed in another independent cohort (cohort 2) including 58 breast cancer patients (Supplementary Fig. 8f, g). *A. muciniphila* and butyric acid levels in fecal were negatively associated with HADS score in cohort 2 breast cancer patients (Supplementary Fig. 8h, i). A significant association was also discovered between fecal *A. muciniphila* abundance and fecal butyric acid levels in cohort 2 breast cancer patients (Supplementary Fig. 8j).

Next, cohort 1 was divided into two groups, including butyric acid in serum low (concentration ≤ 16.52 μM) and butyric acid in serum high (concentration > 16.52 μM), the cutoff based on the median of butyric acid levels in patient serum.^43^ The relationship between butyric acid and clinical pathological parameters was then analyzed. This approach showed that low butyric acid in serum exhibited a positive association with tumor stage, distant metastasis, and the specific biomarker CA153 (Table 2).

**Table 2:** Correlation between butyric acid in serum and clinicopathological parameters

Factor	Butyric acid high		Butyric acid low		*P* value
	NO.	%	NO.	%	
All patients	28	49.1	29	50.9	
**Age**, years					0.5
≤54	13	48.4	17	51.6	
>54	15	50	12	50	
**Tumor size**, cm					0.7
≤1	13	44.8	16	55.2	
>1	15	53.6	13	46.4	
**Node status**					1
Negative	9	50	9	50	
Positive	19	48.7	20	51.3	
**Distant metastasis**					0.02
Negative	21	63.6	12	36.4	
Positive	7	29.2	17	70.8	
**Stage**					0.02
I or II	21	63.6	12	36.4	
III	7	29.2	17	70.8	
**ER status**					1
Negative	6	60	4	40	
Positive	22	46.8	25	53.2	
**PR status**					1
Negative	5	45.5	6	54.5	
Positive	22	48.9	23	51.1	
Missing	1				
**HER2 status**					1
Negative	3	42.9	4	57.1	
Positive	23	47.9	25	52.1	
Missing	2				
**Ki67 status**					1
≤0.3	15	53.6	13	46.4	
>0.3	10	50	10	50	
Missing	3		6		
**CA153**, U/mL					<0.0001
≤9.48	21	72.4	8	27.6	
>9.48	7	25	21	75	

Statistical significance was determined Pearson’s Chi-squared (*χ*^*2*^) test and Fisher’s Exact Test (ER status and HER2 status)

Protein expression of ZFP36, LRP5, β-catenin and stemness factor NANOG in tumor samples was then analyzed. We found that high serumal level of butyric acid exhibited positive correlation with high expression of ZFP36 and low expression of LRP5, β-catenin and NANOG (Fig. 6f–j). In addition, HADS scores were negatively correlated with ZFP36 expression and positively related with LRP5, β-catenin and NANOG expression in cohort 1 breast cancer patients (Supplementary Fig. 8k-n). Moreover, *A. muciniphila* abundance exhibited positive correlation with ZFP36 expression and negative correlation with expression of LRP5, β-catenin and NANOG (Supplementary Fig. 8o–r). Furthermore, ZFP36 expression represented negative association with LRP5/β-catenin expression, while LRP5 level represented positive association with expression of β-catenin and NANOG (Supplementary Fig. 8s–v). Consistently, tumors from breast cancer patients with low butyric acid displayed lower *ZFP36* mRNA and higher *LRP5*/*NANOG*/*SOX2*/*Ki67* mRNA compared with patients with high butyric acid (Fig. 6k, l and Supplementary Fig. 8w–y). Furthermore, lower ZFP36 protein level and higher protein levels of LRP5, β-catenin, NANOG were observed in 5 tumor tissues rather than adjacent normal tissues (Fig. 6m). In addition, PROGgeneV2 analyses indicated that high expression of ZFP36 predicted a good overall survival in breast cancer patients (Fig. 6n). In contrast, high LRP5 and β-catenin expression indicated a poor overall survival (Fig. 6o, p). These results suggest that *A. muciniphila* and butyric acid are promising anti-tumor biomarkers among breast cancer patients with negative mood.

## Discussion

**Fig. 7:**
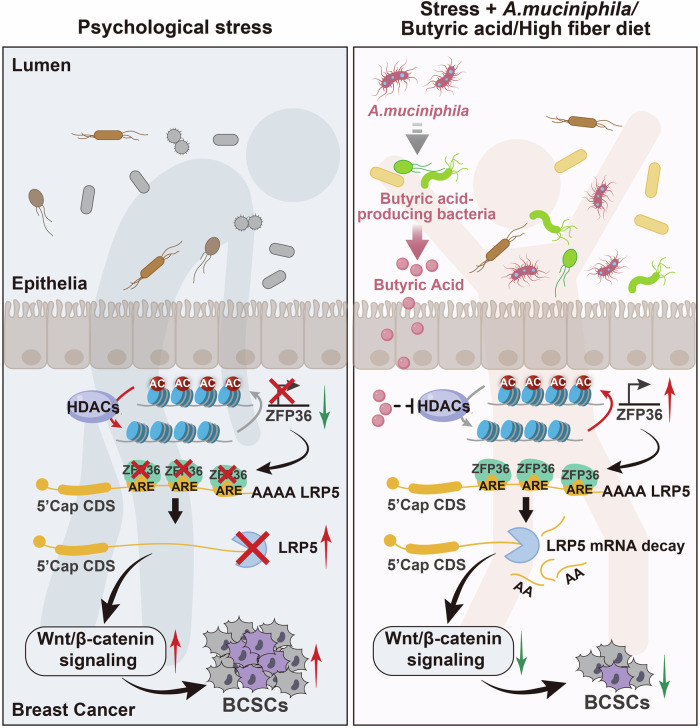
Working model. Psychological stress alters gut microbial community and SCFA profiles during tumor development. Administration of *Akkermansia muciniphila*, butyrate or high-fiber diet markedly reverses stress-caused cancer stemness and anxiety-like behavior. Butyrate transactivates ZFP36 to accelerate LRP5 mRNA decay, thereby blocking Wnt/β-catenin signaling pathway to attenuate cancer stem-like traits. Part of the elements in this figure generated from BioRender (BioRender.com)

Stress-related depression and anxiety have become independent indicators of cancer recurrence and survival.^44^ Here we report that gut microbiota is essential for psychological stress to promote the stem-like properties of breast cancer cells. Using metagenomic sequencing, we discovered that psychological stress has a massive and diverse effect regarding gut microbes. The major effect of psychological stress is to reduce fecal *A. muciniphila* and its metabolite butyrate, leading to a direct promotion of tumor growth and cancer stemness. Importantly, supplementation with *A. muciniphila*, butyrate or a butyrate producing high-fiber diet significantly reverses both psychological stress-induced tumor growth and stemness. Mechanistically, we report that butyrate activates expression of ZFP36 to promote *LRP5* mRNA decay. This causes LRP5 downregulation and suppression of WNT/β-catenin activity, attenuating cancer stemness and tumor growth. Clinical data supports that the reduction of *A. muciniphila* and butyrate levels exhibited correlation among tumoral LRP5/β-catenin expression, anxiety and poor prognosis, indicating a promising role of microbial therapy in stress-related breast cancer for both treating and diagnosing breast cancer (Fig. 7).

Previous reports reveal the critical role of microbiota in promoting tumorigenesis by regulating DNA damage, cytokine production, immune modulation and metabolism disruption.^45^ In addition, microbiota dysbiosis can be induced by psychosocial factors and promote the development of multiple diseases.^46,47^ Moreover, the anti-depressive herbal formula, Xiao-Chai-Hu-Tang, reverses chronic stress-induced microbiota dysregulation leading to an inhibition of tumor progression.^15^ These findings suggest a close association between microbiota and psychological stress-regulated malignacy. Nevertheless, how gut microbiota drives psychological stress-induced tumor development remains poorly understood. Our findings demonstrate that cancer stem-like traits and tumor growth caused by psychological stress are abrogated by microbiota depletion in both germ-free mice and Abx-treated mice. Collectively, these findings clearly support the emerging concept that gut microbiota mediates psychological stress-induced tumorigenesis and cancer stemness.

The impact of gut microbiota on tumors is a comprehensive effect of both probiotics^48^ and pathogenic bacteria.^49^ For example, probiotic *Bifidobacterium* facilitated a subset of CD143^+^ cancer-associated fibroblasts via activating Wnt/β-catenin-GAS1 axis to exhibit tumor suppressive effect.^50^ While pathogenic *F. nucleatum* secretes succinic acid to dampen therapy sensitivity to PD-1 antibodies by impairing CD8^+^ T cell activity.^49^ Our metagenomic sequencing analysis depicted that psychological stress significantly rewires gut microbiota composition by shifting it towards a pathobionts-dominant profile, indicating psychological stress might induce the decrease of probiotics and the increase of pathogenic bacteria promoting cancer progression. Intriguingly, we observed that *A.muciniphila* abundance was dramatically reduced in stress conditions (Supplementary Fig. 3a). Similar with the previous study,^51^ we found that supplement of *A. muciniphila* inhibits breast cancer progression in non-stress conditions. Furthermore, oral gavage with *A. muciniphila* reverses psychological stress-induced tumor growth (Fig. 2g, h) as well as anxiety-like behaviors (Supplementary Fig. 3b, c). As germ-free mice lack the entire microbial community^52^ including the probiotics *A.muciniphila* and other pathogenic bacteria, germ-free mice display little effect on tumor growth and dramatically block the oncogenic effect of psychological stress (Fig. 1c), which is consistent with a recent study.^53^ Our results demonstrate that distinguishing gut microbes as the specific regulator mediates diverse psychological stress-induced cancer phenotypes.

A recent study uncovers that *A. muciniphila* facilitates proliferation of Lgr5^+^ intestinal stem cells to support epithelial development.^54^ However, the effect of *A. muciniphila* in regulating cancer stemness is less understood. Here we show that administration of *A. muciniphila* reverses psychological stress-induced expression of the stemness biomarkers and psychological stress-enhanced sphere formation capacity, establishing a potent function of *A. muciniphila* in dampening cancer stemness under psychological stress. In addition, psychological distress attenuates tumor-infiltrating CD8^+^ T cells function by decreasing *Blautia* and acetic acid.^53^ Interestingly, we find that *A. muciniphila* reverses stress-induced CD4^+^ T cell proliferation and differentiation of Th2, Treg cells, while rescuing stress-inhibited CD4^+^ Th1 cell differentiation (data not shown). The detailed mechanisms of *A. muciniphila* in regulating CD4^+^ T cells mediate psychological stress-induced tumor development is worth to further exploration in the future. In clinical practice, *A. muciniphila* has been shown to improve cancer treatments in various types of cancer.^55^ For instance, *A. muciniphila* in stools is linked to elevated objective response rates of immune checkpoint inhibitors and overall survival in lung cancer patients.^56^ Our study demonstrates important clinical relevance by showing that low fecal *A. muciniphila* is associated with high cancer stemness, advanced anxiety states of breast cancer patients. These findings reveal that *A. muciniphila* act as a promising clinical biomarker for breast cancer and *A. muciniphila* transplantation suggests a potential inervation for breast cancer patients with negative mood.

Accumulating studies demonstrated the ability of SCFAs to suppress cancer development by inhibiting proliferation as well as inducing apoptosis and differentiation of cancer cells.^57^ Here we find that butyrate levels are significantly reduced in the feces, serum, and tumors under psychological stress and are significantly associated with *A. muciniphila* abundance. In addition, butyrate is an important regulator in psychological stress-induced pathology. For instance, elevated butyrate in the brain promotes the microglia-secreted anti-inflammatory factors and averts mental stress-triggered neurogenic abnormalities.^58^ Yet how butyrate regulates stress-induced cancer progression is much less understood. Our findings show that supplement of butyrate reverses stress-induced tumor growth as well as cancer stemness. In addition, butyrate triggers CD8^+^ T cells activity to enhance efficacy of PD-1 antibody.^59^ We further find that the capacity of butyrate blocking tumor growth under psychological stress in immune-deficiency mice is less than the effect in immune-competent mice (data not shown), suggesting butyrate might regulate anti-tumor immunity in mediating stress-induced cancer progression. Since dietary fiber is the major source of microbiota-derived butyrate,^30^ we developed and fed a high-fiber diet to mice in breast cancer mice under psychological stress to further verify the anti-tumor effect of butyrate from the diet. Our findings demonstrate that the high-fiber diet significantly reversed stress-promoted tumorigenesis and cancer stemness. In breast cancer patients, we find that butyrate level exhibits positive correlation with *A. muciniphila* abundance in fecal samples and is negatively correlated with anxiety states and poor prognosis. Thus, a butyrate-producing high fiber diet provides an intervention approach for cancer patients experiencing bad mood.

LRP5 facilitates tumorigenesis as a co-receptor that initiates WNT/β-catenin activation,^60^ which enhances the maintenance of cancer stem cells.^61^ Our results demonstrate that LRP5 and β-catenin are markedly downregulated in butyrate-treated cells. Moreover, the butyrate suppression of LRP5 inhibits β-catenin expression, thus reducing breast cancer stemness. As such, investigating the molecular mechanism of butyrate regulating this newly defined LRP5 target will provide a theoretical foundation and novel therapeutic strategies aimed at removing cancer stem cells. Inhibition of nuclear import of elav-like protein 1 (HuR) mediates *LRP5* mRNA stabilization in the cytoplasm of hepatocellular cancer cells, leading to reduced liver tumorigenesis.^35^ However, the potential mechanism by which LRP5 is regulated in CSCs remains elusive. Here, we advance this knowledge by demonstrating that butyrate downregulates LRP5 expression by accelerating mRNA decay without disturbing transcription. Butyrate is reported to promote the expression of ZFP36, an RNA-binding protein that mediates RNA decay.^62^ Similarly, we found an elevated expression of ZFP36 induced by butyrate treatment and further demonstrated that upregulated ZFP36 binds to the AREs on *LRP5* mRNA to facilitate *LRP5* mRNA decay. In contrast, silencing of *ZFP36* significantly rescues butyrate-restricted Wnt/β-catenin signaling and cancer stemness. Collectively, these new data uncover a novel mechanism by which butyrate regulates *LRP5* mRNA decay and cancer stemness.

Collectively, our data demonstrate that psychological stress-induced breast cancer stemness is mediated by gut microbiota. Psychological stress markedly alters gut microbiome composition, especially by reducing both *A. muciniphila* abundance and butyric acid production. Supplementation of *A. muciniphila* and butyrate dramatically reverses stress-enhanced carcinogenesis and cancer stemness. Furthermore, we uncover a novel mechanism of butyrate attenuating cancer stemness by accelerating *LRP5* mRNA decay via ZFP36. Clinically, we identify *A. muciniphila* and butyrate as potential anti-tumor biomarkers for psychological stress-related breast cancer. Importantly, *A. muciniphila* supplementation as well as a butyrate-producing high fiber diet provide promising intervention strategies for cancer patients with mental stress.

## Materials and methods

### Study approval

Germ-free mice program was carried out under the approval of the Institutional Animal Care and Use Committee (IACUC) of Gempharmatech Co., Ltd (AP#: GPTAP20220225-3), and the experiment was carried out under the supervision of Gempharmatech Co., Ltd IACUC. All SPF mice experiments were conducted in accordance with the Dalian Medical University guidelines for animal care, and all SPF mice procedures were authorized by the IACUC of the Dalian Medical University (Certificate number AEE19040). All patients gave written informed consent and agreed that their samples could be used for clinical research. The Ethics Committee of the Second Affiliated Hospital of Dalian Medical University (No. 2019-028) approved the research protocol.

### Cell culture

Human breast cancer cell lines BT549 (HTB-122), SK-BR-3 (HTB-30), MDA-MB-231 (HTB-26), and the human embryonic kidney epithelial cells HEK293T (CRL-3216) were purchased from the American Type Culture Collection (ATCC). The murine breast cancer Py8119 cell line was donated by Professor Suling Liu’s lab (Fudan University, Shanghai, China). MDA-MB-231 cell line was maintained in Leibovitz’s L-15 Medium (Gibco, 11415064) with 10% fetal bovine serum (FBS; Gibco, 10270). SK-BR-3 cell line was maintained in McCoy’s 5a Medium (Gibco, 16600082) with 10% FBS. BT549 cell line was cultured in RPMI-1640 (Gibco, C11875500BT) with 10% FBS. Py8119 cell line was maintained in F12K (HyClone, SH30526.01) with 10% FBS. HEK293T cells were maintained in DMEM (Gibco, C11995500BT) with 10% FBS. MDA-MB-231 cells were grown in a 37 °C incubator lacking CO_2_, other cells were cultured at 37°C in a humidified incubator containing 5% CO_2_. Anti-mycoplasma reagent SaveIt (Hanbio, HB-SV1000) and 0.1% penicillin-streptomycin (Thermo, 15140122) were added to all media. STR profiling was performed to authenticate cell lines. Cell culture plates and Petri dishes were purchased from NEST Biotechnology Co. Ltd. (Wuxi, China) and Jet Bio-Filtration Co., Ltd (Guangzhou, China).

### Mice

Acquired from GemPharmatech (Nanjing, China), Germ-free (GF) C57BL/6J mice were housed in special plastic isolators designed to house germ-free rodents in GemPharmatech. FVB-MMTV-PyMT mice were obtained from the laboratory of Professor Suling Liu’s lab (Fudan University, Shanghai, China). BALB/c mice and NOD/SCID mice were obtained from Charles River (Beijing, China). C57BL/6J mice were purchased from GemPharmatech. All mice except those that were GF were raised in specific pathogen-free (SPF) feeding environment. At the beginning of experiments, mice were five to seven weeks old. Mice were raised at 50% ± 10% relative humidity, 24 ± 2 °C and a light-dark cycle (strict 24 h: light on 08:00, light off 20:00). Water and food were supported ad libitum.

### Murine breast tumor mouse models

1. Spontaneous breast tumor model
  Stress treatments were performed on mice at seven weeks of age, with breast cancers spontaneously formed by week eight. Body weights and tumor volumes were measured every two days. Behavioral tests were conducted three weeks after tumors formed. Finally, tumor-bearing organs were harvested after breast cancers spontaneously formed for 25 days. Tumor volumes were assessed by measuring the perpendicular dimensions with calipers. The estimated formula = 0.5 × a × b^2^ (a was tumoral long diameter and b was tumoral short diameter)
2. Subcutaneous syngeneic tumor model
  Suspensions of Py8119-pLVX-MCS-Luc2 or Py8119 tumor cells were prepared for implantation (1 × 10^5^ cells in PBS/Matrigel [1:1]). Cell mixture resuspended in 50 μl Matrigel (Corning, 354230) and 50 μl PBS was subcutaneously injected into both SPF and GF mice (C57BL/6J).
3. MDA-MB-231 xenograft tumor model
  MDA-MB-231 cells (1 × 10^6^ cells per mouse) were orthotopically injected into female NOD/SCID mice. The tumor sizes were assessed by measuring the perpendicular dimensions with a caliper every other day. Xenografted tumors were immediately dissected to take photos at the ethical endpoint.
4. Orthotopic syngeneic tumor model
  Py8119-pLVX-MCS-Luc2 or Py8119 tumor cells (1 × 10^5^ cells per mouse) were orthotopically injected into the murine fat pads (C57BL/6J). Vernier calipers were used to measure the size of tumors and calculate the volumes. Tumors were immediately dissected, measured and photographed at the ethical end point.

4T1 tumor cells (1 × 10^5^ cells per mouse) were orthotopically transplanted into murine fat pads (BALB/c). Vernier calipers were used to measure the size of tumors and calculate the volume as described above. Tumors were immediately dissected, measured and photographed at the ethical end point.

### Chronic restraint stress (CRS) model

Mice with CRS were confined for 6 hours (from 10: 00 to 16: 00) each day lasting 28 days (from Day -7 to Day 21) in a 50 ml tapered tube with holes to constrain free movement and rotation and provide adequate air circulation until the ethical endpoint. All stressed mice underwent restraint pretreatment for seven days to acclimate prior to tumor transplantation. Food and water were removed from the cages of Ctrl mice from 10: 00 to 16: 00.

### Behavioral Paradigms

Mice behavioral tests started on the second day following the final stressed management. All mice were placed in the test room 1.5 h prior to initiation of tests to allow acclimation to the surrounding. Animal movement was recorded with a camera and analyzed with software (Xeye Aba, Beijing MacroAmbition S&T Development Co., Ltd.). Following each test, all testing equipment was disinfected with 75% ethanol to remove any residual odors, urine, and feces. The detailed methods of open-field test and elevated plus maze test were described previously.^3^

### Pharmacological studies in mice

1. Antibiotic treatment
  An antibiotic mixture was given to mice *ad libitum* with sterilized fresh drinking water every two days. This mixture contained 100 μg/ml neomycin (Sigma, N6386), 50 μg/ml streptomycin (MCE, HY-B0472), 100 μg/ml ampicillin (Sigma, A9518), 50 μg/ml vancomycin (Sigma, V2002), 100 μg/ml metronidazole (Sigma, M3761), 1 mg/ml bacitracin (MCE, HY-B0278), 125 μg/ml ciprofloxacin (Sigma, PHR1167), and 100 μg/ml ceftazidime (Solarbio, C9731).
2. Sodium butyrate oral gavage
  We randomly assigned mice to the following experimental groups: normal saline (0.9% w/v), sodium butyrate (NaBu) (sigma, B5887) treatments. In the NaBu treatment group, mice were orally gavaged with NaBu solution (50, 100, 200 mg/kg) per day for 21 days, which was dissolved in saline and filtered through a 0.22 μm membrane. Equal volume of sterilized saline was added to control mice.
3. High fiber diets
  We randomly assigned mice to two diets: a diet added with inulin as a source of fiber or a control diet (mFD/mCD; TP 23300-X1/LAD 3001 G, Trophic Animal Feed High-tech, China). Both mCD and mFD diets contained 19.4% kcal from protein, 63.9% kcal from carbohydrates, and 16.7% kcal from fat. All mice received mCD for the first week of the experiment, after which mice were given either the mCD or mFD diets. During the procedure, murine body weights were measured every two days. Feed disappearance was measured to confirm that mice consumed a similar amount of feed between the mCD group and mFD group.
4. Akkermansia muciniphila supplementation
  To evaluate the tumor suppressive role of *Akkermansia muciniphila* (*A. muciniphila* strain #1, AKK) (ATCC number: BAA-835) and (*A. muciniphila* strain #2, AKK-2) (DSMZ number: DSM26127) supplementation on tumor growth, *A. muciniphila* was first anaerobically incubated in media with brain heart infusion (BHI) at 37°C. The bacterial cultures were then incubated with 25% glycerol in saline and adjusted to a final concentration of 1 × 10^10^ colony-forming units (CFU)/ml. Cultures were immediately frozen and stored at -80°C. Before gavage, the glycerol stock solution was thawed under anaerobic conditions and resuspended in anaerobic normal saline to a concentration of 3 × 10^8^ CFU in 200 μl and each mouse was treated with 200 μl. As the bacterial associated with stress, *Alistipes shahii* (ATCC number: BAA-1179) was utilized as negative control. Oral gavage with the 200 μl *A. muciniphila* (3 × 10^8^ CFU) suspension in mice once a day for 21 days. For dead-*A. muciniphila* (heat-killed) experiments, 3 × 10^8^ CFU bacterial *A. muciniphila* per mice were heated at 95°C for 2.5 h before gavage.

### In vivo bioluminescence imaging

The Py8119-pLVX-MCS-Luc2 breast cancer cell line was subcutaneously or orthotopically injected into mice. These cells express luciferase gene as an optical indicator of tumorigenesis, which allowed for in vivo bioluminescence imaging as the tumor grows. To do so, mice were anesthetized under isoflurane inhalation followed by i.p. administration of the substrate fluorescein. After a period of 15 minutes, a three-dimensional multimodal imaging equipment (Carestream In-Vivo Imaging System FX PRO, Carestream Health) was employed in vivo for luminescence imaging.

### Plasmid construction

Human LRP5 CDS regions were produced through PCR augmentation and then inserted into the pCDH expression vector via subcloning to create the pCDH-LRP5 plasmid. LRP5 3’UTR were produced through PCR amplification and inserted into the psiCHECK2 vector to create the psiCHECK2-LRP5-3’UTR-WT plasmid. Then psiCHECK2-LRP5-3’UTR (Mut1, Mut2 and Mut1 + 2, predicted AU-rich element mutant) plasmids were produced by performing site-directed mutagenesis by PCR. Detailed information of primer sequences for establishment of plasmids is provided in Supplementary Table 3.

### Transfection, package of lentivirus, and establishment of stable cell lines

Plasmids or siRNA (GenePharma, Suzhou, China) were transiently transfected with Lipofectamine 3000 (Invitrogen, L3000015) described by manufacturer’s protocol. The second-generation packaging system plasmids psPAX2 (Addgene plasmid, 12260) and pMD2.G (Addgene plasmid, 12259) were used for lentiviral packages in HEK293T cells. Lentiviral plasmid pCDH-LRP5 or pLVX-MCS-Luc2 (Shanghai Haijihaoge Biotechnology, HH-LUC-106) as well as pMD2.G and psPAX2 were transiently co-transfected. After transfection with 48 and 72 h, supernatant virus was collected and titer was measured. For generation of Py8119 (pLVX-MCS-Luc2), SK-BR-3 (pCDH-LRP5), MDA-MB-231 (pCDH-LRP5) stable cell lines, cells were transduced by viruses and screened by puromycin (2 μg/ml, Sigma, P8833). All siRNAs are provided in Supplementary Table 4.

### Dual-luciferase reporter assay

A dual-luciferase reporter gene assay was conducted by a detection kit following the manufacturer’s protocol (Promega, E1910). psiCHECK2-LRP5-3’UTR (WT, Mut1, Mut2 and Mut1 + 2) and siRNAs or other plasmids were co-transfected into HEK293T. Twenty-four hours later, Dual-Luciferase Assay System was used for relative luciferase activity tests.

### RNA stability assay

SK-BR-3 cells and MDA-MB-231 were cultured with NaBu for 12 h or transfected with siRNA plus that was cultured with NaBu for 12 h. After treatment with actinomycin D (5 μg/ml ActD, Sigma, SBR00013), cells were collected at 0, 2, 4 and 6 h. RT-qPCR was then performed to evaluate RNA stability.

### ALDH^+^ cell staining

ALDEFLUOR reagent was used to measure cell aldehyde dehydrogenase (ALDH) activity (STEMCELL Technologies, 01700) following the manufacturer’s protocol. In brief, 1 ml of ALDEFLUOR assay buffer containing testing cells mixed by 5 µl of activated ALDEFLUOR reagent, and immediately transferred into 500 µl of assay buffer that held 2 × 10^5^ cells and into the negative control tubes containing ALDEFLUOR DEAB reagent (5 µl). All samples were incubated at 37°C for 0.5 h. Cells were measured by the flow cytometer (Beckman Coulter, CytoFLEX) to determine the fluorescence intensity of ALDH^+^ cells.

### Western blotting

The detailed procedure was described in our previous study.^18^ Antibodies used for western blotting were: LRP5 (Proteintech, 24899-1-AP), Sox2 (Santa Cruz Biotechnology, sc-365823), ZFP36 (Proteintech, 12737-1-AP), β-catenin (Proteintech, 51067-2-AP), NANOG (Gene Tex, GTX100863), Lamin B1 (Proteintech, 66095-1-lg), Phospho-GSK3β (Ser9) (Proteintech, 67558-1-lg), GSK3β (Proteintech, 67329-1-Ig), Histone H3 (Proteintech, 17168-1-AP), Acetyl-Histone H3-K9 antibody (ABclonal, A21107) and beta Actin (Proteintech, 66009-1-lg). The prestained protein marker was obtained from Thermo Fisher Scientific (26617).

### Quantitative PCR (qPCR) analysis

To validate bacterial abundance, fecal DNA was subjected to qPCR with specified primers. PCR reactions contain template DNA (4 μl, 25 ng/μl), SYBR Green qPCR mix (5 μl, ABclonal, RK21203), nuclease-free water (0.5 μl), and specific primer (0.25 μl, 20 μM). Values were normalized to Eubacteria. For quantitative reverse transcription PCR (RT-qPCR), total RNA was isolated from cell lines or tissue samples using TRIzol reagent (Invitrogen, 15596018) following the manufacturer’s protocol. The cDNAs were synthesized by the Evo M-MLV RT kits (Accurate Biotechnology, AG11705) and qPCR was addressed with SYBR Green qPCR mix (ABclonal, RK21203). *ACTB* as the internal control was used to normalize RNA levels. All primers are listed in Supplementary Table 5.

### Sphere formation assay

Suspended single cells were prepared and plated in 24-well ultra-low attachment plates (Corning, 3473) and cultured using serum-free DMEM/F12 medium (Gibco, C11330500BT) with 20 ng/ml epidermal growth factor (Sigma, E9644), 1% methylcellulose (R&D Systems, HSC001), 20 μg/ml B27 (Gibco, 17504044) and 20 ng/ml basic fibroblast growth factor (Peprotech, 100-18B). Every other day, cultures were replenished with sphere medium. The plate was placed in 37°C humidified incubator with 5% CO_2_ for about two weeks until spheroids were formed. The wells containing spherical cells were photographed for and subsequently measured.

### Extreme limiting dilution assay (ELDA)

Cells were seeded onto 96-well ultra-low attachment plates (Corning, 3474) containing sphere medium as described above at a density of 2, 5, 10, 20, 50, and 100 cells/well (24 wells per cell density). The number of sphere forming wells within each group was calculated after 7 days in the ELDA website.^63^

### RIP assay

MagnaRIP kits were utilized following the manufacturer’s instructions (Millipore, 17-700). Cells were incubated on ice for 0.5 h with RIP lysis buffer. They were subsequently incubated with magnetic protein A-protein G beads as well as 5 µg of either ZFP36 antibody (Proteintech, 12737-1-AP) or normal IgG antibody (Millipore) for negative control at 4 °C for 3 h. The beads were subjected to three washes with protease K buffer (55 °C, 0.5 h). Subsequently, the co-precipitated RNAs were isolated using Enol: Chloroform: Isoamylol (pH < 5.0; Solarbio life science, P1011) and analyzed by RT-qPCR.

### ChIP assay

Chromatin immunoprecipitation was conducted following the manufacturer’s protocol by ChIP Kits (Active Motif, 53008). Briefly, cells (2 × 10^7^) were treated with fixation solution for 8 min at room temperature on a shaker to facilitate the cross-linking of histones to DNA and washed in ice-cold PBS. Glycine was added to the plate for 5 min to terminate the fixation reaction. Cells were subsequently harvested by scraping solution containing PMSF (0.6 mM) and then centrifuged (10 min, 3000 × *g*, 4 °C). Cells were resuspended in pre-chilled lysis buffer (1 ml) and incubated for 0.5 h at 4 °C. and gently homogenized 10 times on ice using a pre-chilled dounce homogenizer to promote the release of nuclei. DNA was fragmented using a sonicator, then centrifuged (10 min, 14,500 × *g*, 4 °C). A 10 μl aliquot was carefully collected as “input DNA”. The left chromatin was immunoprecipitated with protein A/G magnetic beads (Invitrogen, 10001D) and Acetyl-Histone H3-K9 antibody (2 μg, ABclonal, A21107) or a negative control IgG antibody (Thermo Fisher Scientific, 31460) at 4 °C for 4 h. Beads were washed by ChIP buffer I and II, then resuspended in elution buffer AM2 (50 μl). Chromatin was eluted using reverse crosslinking buffer (50 µl) and incubated with proteinase K (2 μl) for 2 h. DNA was purified by adding 2 μl Proteinase K stop solution, followed by centrifugation, and used for qPCR.

### Cytoplasmic and nuclear protein extraction

Cytoplasmic component in cell pellets were obtained by a hypotonic buffer (3 mM MgCl_2_, 20 mM Tris-HCl pH 7.4, 10 mM NaCl,) with a protease inhibitor cocktail (MCE, HY-K0010), NP-40 (0.9%) was then added followed by vertexing 10 sec and centrifuged at 4 °C, 3,000 × *g*, 10 min. Supernatants were moved into a fresh tube and the remaining pellets were washed 3 times with hypotonic buffer followed by discarding the supernatants. Nucleoprotein was isolated with specialized nuclear extraction buffer (1% TritonX-100, 100 mM Tris-HCl, pH 7.4, 100 mM NaCl, 1 mM EGTA, 20 mM Na_4_P_2_O_7_, 1 mM NaF, 0.1% SDS, 2 mM Na_3_VO_4_) and centrifuged at 4 °C, 14,500 × *g*, 30 min. The nuclear protein fraction was used for further analysis.

### Immunofluorescence (IF)

Cells were fixed using 4% paraformaldehyde, permeabilized with 0.5% TritonX-100, and subsequently treated by blocking buffer (2% BSA; Sigma, V900933) for 2 hours. Cells were incubated by β-catenin antibody (Proteintech, 51067-2-AP) overnight at 4°C, and secondary antibodies (1:200 with 1% BSA in PBS) at room temperature for 1 h. Samples were counterstained with 4’, 6-Diamidino-2-phenylindole dihydrochloride (DAPI) (1:2000 in PBS) (Sigma-Aldrich, D9542) and mounted on glass slides with anti-quenching medium. Images were acquired and analyzed using a confocal microscope (Leica, TCS SP5II).

### Enzyme-linked immunosorbent assay (ELISA)

The serum cortisol levels (Cloud-Clone, CEA462Ge) in 57 breast cancer patients were measured using an ELISA kit, following the manufacturer’s protocol. Add 50 μl of the standard dilutions, blank, and samples to the respective wells. Afterward, add 50 μl of Detection Reagent A and incubate at 37°C for 1 h. Remove the solution by aspiration and rinse each well 3 times with 350 μl of Wash Solution. Add Detection Reagent B working solution (100 μl) and incubate at 37 °C for 30 min. Next, repeat the wash step 5 times, then add 90 μl of Substrate Solution and incubate at 37°C for 10 min without light. The optical density at 450 nm wave-length was measured after the addition of 50 μl of Stop Solution, The standard curve was established, and the concentrations were determined based on this curve.

### Immunohistochemical (IHC) staining and analysis

According to standard procedures, tissue specimens were first embedded in paraffin, followed by deparaffinization and rehydration. Antigen retrieval was performed with citric acid (pH 6.5) for 3 min. 3% hydrogen peroxide in H_2_O and 10% goat serum were used to block endogenous catalase. The NANOG (1:100, Gene Tex, GTX100863), Ki67 (1:200, Abcam, ab15580), ZFP36 (1:150, proteintech, 12737-1-AP), LRP5 (1:150, proteintech, 24899-1-AP), β-catenin (1:200, Proteintech, 51067-2-AP) antibodies were used overnight at 4°C, followed by a 1-hour incubation with HRP peroxidase-bound secondary antibody. Specimens were stained by DAB (ZSGB-BIO, ZLI-9018) and washed with water to stop the reaction. Nuclei were identified with hematoxylin staining (Coolaber, SL7050) for 4-6 min and rinsed with water. Finally, Specimens were dehydrated and sealed with neutral resin. HRP peroxidase-bound secondary antibodies were derived from IHC kit (ZSBIO, pv-9002). Six random areas were chosen to measure the positive levels in each tumor specimens and quantified by ImageJ.

### Hematoxylin-eosin (HE) staining and analysis

HE staining was performed to assess the proliferation of tumor cells in mouse tissue. Sections of Tumor tissue paraffin were first immersed in xylene I, followed by xylene II, anhydrous ethanol I, anhydrous ethanol II and 75% ethanol, then rinsed with water. Finally, sections were dehydrated, mounted with neutral gum, and then examined under a microscope for image capture. Six randomly selected areas were picked out to measure the positive levels in every section, and quantified by ImageJ.

### Metagenomics sequencing and data analysis

DNA from various samples (Feces of SPF-Ctrl and SPF-Stress mice, Feces of Ctrl, Stress, AKK, Stress+AKK mice, Feces of HFD and Stress+HFD mice, Feces of 57 breast cancer patients in cohort 1) was extracted by means of a Stool DNA Kit (Omega, D4015-02) in accordance with the manufacturer’s protocols. DNA library construction and data analysis were performed by BIOZERON (Shanghai, China) for the 57 patients’ fecal metagenomics data of cohort 1 and LC-Bio (Hangzhou, China) for the mouse fecal metagenomics data.

### RNA-seq

Total RNA was extracted from SK-BR-3 cells treated with NaBu for 48 h using TRIzol reagent (Invitrogen, 15596018) for RNA-seq. The following processes including the establishment of RNA libraries sequenced on the Illumina Hiseq platform, alignment of RNA-seq data, gene expression, read count mapping, differentially expressed genes (DEG) analysis, and gene ontology (GO) enrichment analysis were performed by Novogene (Beijing, China) following the manufacturer’s protocol. The detailed procedure was described previously.^18^

### SCFAs analysis of fecal, serum, and tumor samples in mice

1. Sample preparation.
  The internal standard (IS) of 2-ethylbutyric acid was spiked into acetonitrile to a final concentration of 10 μmol/l. For serum, 60 μl acetonitrile with spiked IS was added to 15 μl serum for protein participation. After vortexing and centrifugation, supernatants were injected into gas chromatography vials for further analysis. For feces, 200 μl acetonitrile with spiked IS was added to ~20 mg of fecal samples. Following the addition of clean zirconia grinding beads, homogenization and metabolite extraction was performed twice using a mixed grinding apparatus (RETSCH, MM400) at 20 Hz for 1 min. The mixture was subsequently centrifuged at 4°C, 18,000 × *g*, 10 min, and collected supernatant was used for analysis. For tissue, 200 μl acetonitrile with spiked IS was added to ~50 mg tissue samples. Once homogenization and centrifugation were completed, 60 μl of the supernatant was utilized for GC-MS detection.
2. Gas chromatography-mass spectrometry (GC-MS) analysis.
  Instrumental analysis was carried out using a gas chromatography (Agilent, 5977 A) linked to an MSD mass spectrometer (Agilent, 5977 A), which was fitted with a capillary column (30 m × 0.25 mm i.d., 0.25 μm film thickness; Agilent, DB-FFAP). Helium (99.9995%, Kena Co., Dalian, China) served as the carrier gas, maintained at a constant linear velocity of 40 cm/s. The sample size was 1 μl for the splitless inlet. The program temperature was shown: 50°C initial column temperature for 1 min, then raised to 180°C at a rate of 10°C/min, followed by an increase to 250°C at a rate of 30°C/min, and finally held at 250°C for 2 min. Mass spectrum was acquired in the selected ion monitoring (SIM) mode with a scan range of 35-100. The precursor ions of acetic acid, propionic acid, butyric acid, isobutyric acid, valeric acid, isovaleric acid, hexanoic acid, and 4-methylvaleric acid were included in the acquisition method.
3. Data analysis
  Raw data were imported into MassHunter Workstation software for metabolite extraction and integration. After IS calibration, the peak area of the target compound was projected onto the corresponding standard curve to achieve absolute quantification. Finally, a three-dimensional matrix was generated containing compound names, absolute contents (variables), and sample names (observations).

### SCFAs analysis of human fecal and serum samples

1. Sample preparation.
  Samples were isolated with 80% methanol-water solution (1 ml). We added 2 steel beads, ground, vortex and mixed, then centrifuged (20,000 × *g*, 4 °C) for 15 min. Supernatant (20 μl) was put in a 1.5 ml Ep tube. We added 3-NPH (20 μl) and EDC reagent (20 μl), put into a 40°C water bath for 30 min, added the initial mobile phase solution to 500 μl, vortex and mixed, then took 200 μl into the injection vial for LC-MS/MS analysis.
2. UHPLC-MS/MS analysis.
  The target compounds were separated and quantified with the use of a Thermo Scientific TSQ Altis mass spectrometer with Vanquish liquid chromatograph. The chromatographic separation was conducted on an ACQUITY UPLC BEH C18 column (1.7 μm, 3.0 × 100 mm, Waters). Water (A) and methanol + acetonitrile (1:1) (B) were used as mobile phases for liquid chromatography. The temperature of column was maintained at 40°C. The flow rate of the mobile phase was adjusted to 300 μl/min and the injection volume was 2 μl. UHPLC-ESI-MS was employed for sample introduction and ionization, while low-energy collision-induced dissociation tandem mass spectrometry was conducted in the selective reaction monitoring mode. The vaporizer temperature was 300°C. To improve the working conditions of the source, each standard compound was continuously infused into the ESI source through direct injection using a syringe pump. Collision energy (CE) was adjusted to achieve the highest sensitivity of precursor ion → product ion transition in the SRM scan. The analysis involved monitoring two SRM transitions for each compound: one for quantification (quantifier transition) and the other for confirmation (qualifier transition) of each compound.
3. Data preprocessing.
  In this project, we quantified data using external standard method and the retention time and MRM fragment ions were compared by the methodology of the standards for characterization. During the test, samples with detection concentrations above the range of the standard curve were diluted at appropriate multiples and retested, and the data measured after dilution were used as the quantitative results. The patients’ fecal data of cohort 1 provided by LC-Bio, the patients’ serum data of cohort 1 and the patients’ fecal data from cohort 2 were provided by Key Laboratory of Separation Sciences for Analytical Chemistry, National Chromatographic R&A Center, Dalian Institute of Chemical Physics, CAS.

### Biospecimen collection

In the first cohort (cohort 1), HADS questionnaires, tumor specimens, serum, and stool specimens, were obtained from 57 breast cancer patients receiving examination and treatment at the Second Affiliated Hospital of Dalian Medical University. In the second cohort (cohort 2), stool specimens, HADS questionnaires, and stool samples were obtained from 58 breast cancer patients receiving examination and treatment at the Second Affiliated Hospital of Dalian Medical University. The collection of all specimens adhered to the informed consent policy.

### Statistics

Statistics were made by either GraphPad Prism 6.0 (GraphPad Software Inc.) or SPSS software (version 16.0). Statistical comparisons were performed using one-way ANOVA test, Student’s *t*-test (two-tailed unpaired), or the likelihood ratio test as indicated in Figure legends. The Chao 1 index was calculated to indicate alpha diversity. PCA and PCoA were analyzed using Weighted UniFrac ANOSIM analysis software to establish beta diversity. KEGG Annotation and GO analysis programs were used to identify key metabolic pathways that were altered by psychological stress and analyzed using a two-tailed Mann-Whitney U-test. Tumor growth curve data were analyzed at the ethical endpoint using a two-tailed, unpaired Student’s *t*-test. The association between clinical indicators was evaluated using Pearson’s Chi-squared (*χ*^*2*^) tests or Fisher exact tests, with statistical significance set at *P* < 0.05. Analyses were carried out using R version 3.6.3. Overall survival (OS) was estimated by Kaplan-Meier plots. The significance of differences in survival rates was assessed using the log-rank test. 95% confidence intervals and Hazard ratios were assessed for each variable. Correlations between species abundance and clinical markers were evaluated with Pearson’s rank correlation test.

## Supplementary information

Supplementary Materials

Dataset1

Dataset2

## Data Availability

The data backing the findings on this research can be accessed online. All RNA-seq datasets generated have been uploaded into the Sequence Read Archive (SRA) database. Metagenomics sequencing data of murine fecal material is available at (PRJNA926372) and RNA sequencing data of SK-BR-3 cells is available at (PRJNA924300). Metagenomics sequencing data for fecal samples of clinical patients is available at (PRJNA1188760). Source data are supplied in the current paper.
