# Supplementary material for: Gut dysbiosis conveys psychological stress to activate LRP5/β-catenin pathway promoting cancer stemness
Source: Signal Transduct Target Ther. 2025 Mar 5;10:79. doi: 10.1038/s41392-025-02159-1 (PMC11880501; doi:10.1038/s41392-025-02159-1)
Supplement: Supplementary file 1 — Supplementary Materials [file 41392_2025_2159_MOESM1_ESM.doc]

Supplementary Materials for

Gut dysbiosis conveys psychological stress to activate LRP5/β-catenin pathway promoting cancer stemness

Bai Cui^1,8^, Huandong Luo^1,2,8^, Bin He^1,8^, Xinyu Liu^3,8^, Dekang Lv^1,8^, Xiaoyu Zhang^1^, Keyu Su^1^, Sijia Zheng^3^, Jinxin Lu^1^, Cenxin Wang^1^, Yuqing Yang^1^, Zhuoran Zhao^1^, Xianxian Liu^1^, Xu Wang^1^, Yingrui Zhao^1^, Xiaoshan Nie^1^, Yuanyuan Jiang^1^, Ziyu Zhang^1^, Congcong Liu^1^, Xinyi Chen^1^, Anqi Cai^1^, Zhumeng Lv^1^, Zhihang Liu^1^, Fan An^1^, Yunkun Zhang^4^, Qiulong Yan^5^, Keith W. Kelley^6^, Guowang Xu^3^, Lingzhi Xu^7*^, Quentin Liu^1,2*^ and Fei Peng^1*^

Correspondence to: [pengfei@dmu.edu.cn](mailto:pengfei@dmu.edu.cn) (F.P.); [liuq9@mail.sysu.edu.cn](mailto:liuq9@mail.sysu.edu.cn) [xvlingzhi@sina.com](mailto:xvlingzhi@sina.com) (L.-Z.X.)

**This PDF file includes:**

Figures. S1 to S8

Tables S1 to S5



**Fig. S1. Establishment and validation of psychological stress in mice with breast cancer.** (**a**) Mice were subjected to behavioral tests after completing the chronic stress paradigm. Time spent in the center was analyzed by the open field test (n = 10 mice per group). (**b**) Number of entries in the open arms was analyzed using an elevated plus maze (n = 10 mice per group). (**c**) Body weight changes in specific pathogen free (SPF) and germfree (GF) mice under control (Ctrl) and stress (Stress) conditions (n = 10 mice per group). (**d**) Representative images of cecum in SPF/GF mice. (**e**) Relative *Nanog* and *Sox2* mRNA expression in SPF and GF tumors (n = 3 biological replicates). (**f**) Relative expression of Firmicutes and Actinobacteria in feces of MMTV-PyMT mice (n = 10 mice per group). (**g**) Mice were subjected to behavioral tests after completion of the chronic stress paradigm. Time spent in the center was analyzed in the open field test (n = 6 mice per group). (**h**) Number of entries in open arms was analyzed by the elevated plus maze (n = 6 mice per group). (**i**) Body weight changes in Ctrl and Stress groups treated with antibiotic cocktail (Abx) or H_2_O (n = 6 mice per group). (**j**) Relative *Nanog* and *Sox2* mRNA expression in spontaneous tumors (n = 3 biological replicates). (**k**) Relative Firmicutes and Actinobacteria expression in feces of the Ctrl or Stress groups treated with Abx or H_2_O (n = 8 mice per group). (**l**) Mice were subjected to behavioral tests after completing the chronic stress paradigm. Time spent in center was analyzed by the open field test (n = 8 mice per group). (**m**) Number of entries in open arms was analyzed by using an elevated plus maze (n = 8 mice per group). (**n**) Body weight change in Ctrl and Stress groups treated with Abx or H_2_O (n = 8 mice per group). (**o**) Representative images of cecum in Ctrl and Stress groups with or without Abx. (**p**) Representative microscopic pictures of Hematoxylin-eosin (HE) staining, NANOG and Ki67 immunohistochemical (IHC) staining in tumors (Scale bar, 50 μm). (**q**) Quantitative results of HE and IHC staining from indicated markers in indicated groups (n = 6 representative images). (**r**) Relative *NANOG* and *SOX2* mRNA expression in MDA-MB-231 xenograft tumors (n = 3 biological replicates). Results are represented as mean ± s.d. (**a-c**, **e**, **g-j, l-n,** **q**, **r**) by one-way ANOVA, (**f, k**) by two-tailed, unpaired Student’s *t*-test. *P* values are as indicated.


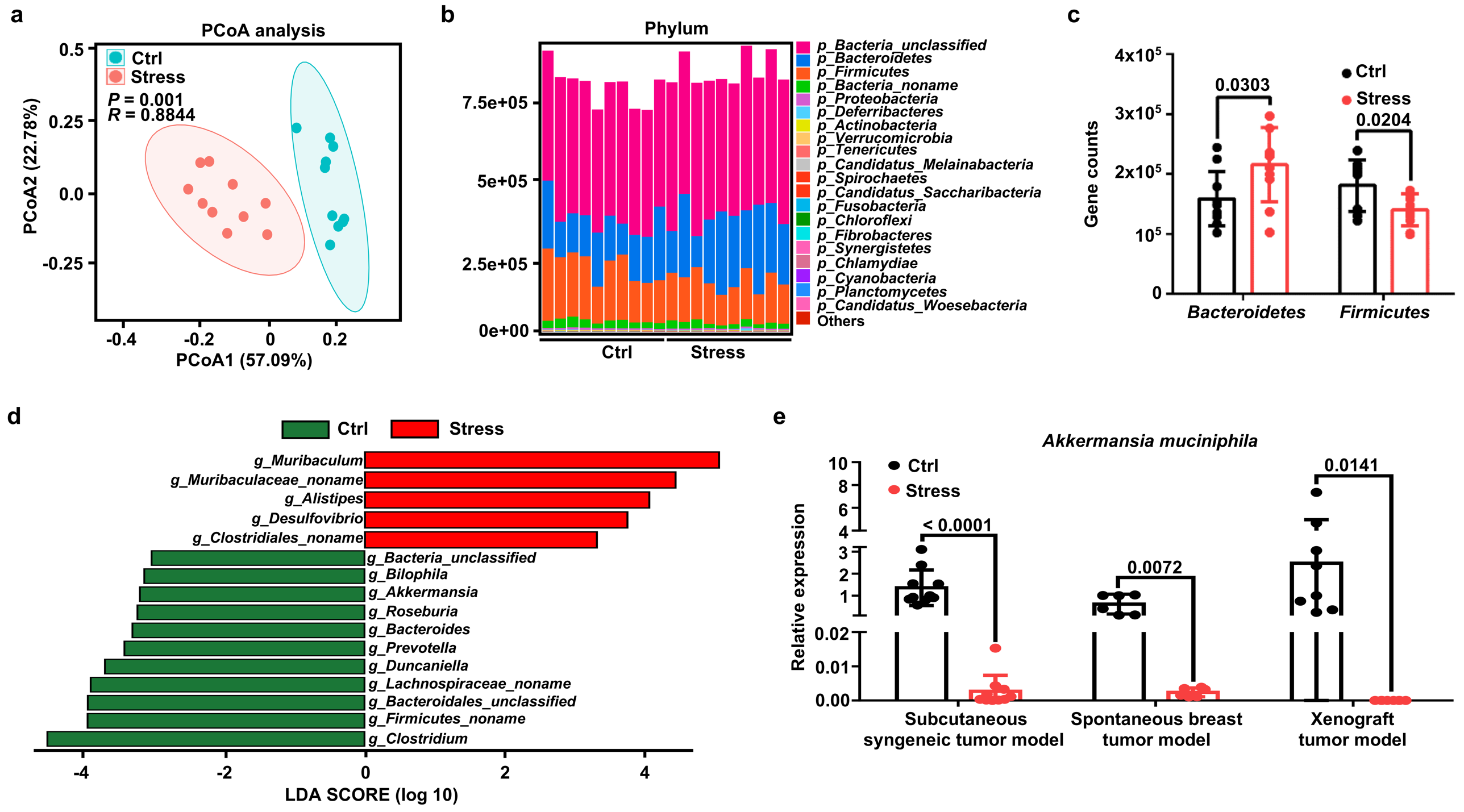
**Fig. S2. Psychological stress modulates the composition of gut commensal bacteria.** (**a**) PCoA of fecal microbiota from the Ctrl and Stress mice inoculated with Py8119 cells (n = 10 mice per group). (**b**) Bar plots displaying taxonomic composition in Ctrl and Stress mice inoculated with Py8119 cells at the phylum level. Relative abundance is plotted for each mouse (n = 10 mice per group). (**c**) Gene counts of Bacteroidetes and Firmicutes in Ctrl and Stress groups (n = 10 mice per group). (**d**) LDA score calculated at the genus level based on the differentially abundant features between the Ctrl and Stress mice inoculated with Py8119 cells. The criteria of feature selection are log10 LDA score > 3. (**e**) Relative *A*. *muciniphila* expression between Ctrl and Stress groups in the subcutaneous syngeneic tumor model (n = 10 mice per group), spontaneous breast tumor model (n = 6 mice per group) and xenograft tumor model (n = 8 mice per group). Results are represented as mean ± s.d. (**a**) by Weighted UniFrac ANOSIM analysis, (**c, e**) by a two-tailed, unpaired Student’s *t*-test. For multiple comparisons, p-values were adjusted using the FDR correction. *P* values are as indicated.



**Fig. S3. Supplementation of *A. muciniphila* reverses psychological stress-induced anxiety-like behavior and cancer stem-like traits.** (**a**) Relative *A. muciniphila* expression in feces of mice (n = 8 mice per group). (**b**) Mice were subjected to behavioral tests after completing the chronic stress paradigm. Time spent in the center was analyzed by the open field test (n = 8 mice per group). (**c**) Number of entries in open arms was analyzed using the elevated plus maze (n = 8 mice per group). (**d**) Body weight changes in Ctrl and Stress groups treated with *A. muciniphila* or saline (n = 8 mice per group). (**e**) Schematic of the Ctrl or stressed BALB/c mice inoculated with 4T1 cells with *A. muciniphila* (AKK) administration. The AKK and Stress+AKK mice groups were treated with 200 μl (3 × 10^8^ CFU) AKK for 21 days (n = 6 mice per group). (**f**) BALB/c mice were subjected to behavioral tests after completing the chronic stress paradigm. Time spent in center was analyzed by the open field test (n = 6 mice per group). (**g**) Number of entries in open arms was analyzed by the elevated plus maze (n = 6 mice per group). (**h**) Body weight changes in Ctrl and Stress groups treated with *A. muciniphila* or saline (n = 6 mice per group). (**i**) Representative tumor image from BALB/c mice treated with AKK or saline in the Ctrl and Stress groups (n = 6 mice per group). (**j**) Tumor volumes curves in Ctrl and Stress mice treated with AKK or saline (n = 6 mice per group). (**k**) Schematic of the Ctrl or stressed C57BL/6J mice inoculated with Py8119-pLVX-MCS-Luc2 or Py8119 cells with another strain of *A. muciniphila* #2 (AKK-2) administration. The AKK-2 and Stress+AKK-2 mice groups were treated with 200 μl (3 × 10^8^ CFU) AKK for 21 weeks days, the bacterial *Alistipes shahii* (*A. shahii*) as a negative control (n = 6 mice per group). (**l**) C57BL/6J mice were subjected to behavioral tests after completing the chronic stress paradigm. Time spent in center was analyzed by the open field test (n = 6 mice per group). (**m**) Number of entries in open arms was analyzed by the elevated plus maze (n = 6 mice per group). (**n**) Body weight changes in Ctrl and Stress groups treated with *A. muciniphila*, *A*. *shahii* or saline (n = 6 mice per group). (**o**) Representative bioluminescence images of Py8119-pLVX-MCS-Luc2 or Py8119 orthotopic tumors in Ctrl and Stress groups treated with AKK-2, *A. shahii* or saline. (**p**) Tumor volumes curves in Ctrl and Stress mice treated with AKK-2, *A. shahii* or saline (n = 6 mice per group). (**q**) Relative *Nanog* and *Sox2* mRNA expression in Py8119-pLVX-MCS-Luc2 orthotopic tumors (n = 3 biological replicates). (**r**) Representative images of spheroids formed by single Py8119-pLVX-MCS-Luc2 tumor primary cell in the Ctrl and Stress groups treated with *A. muciniphila* or saline (left), number of spheres per 300 cells (d > 50 μm) (middle) and distribution pattern of sphere diameter (right) (Scale bars, 100 μm). (**s**) ELDA were performed in Py8119-pLVX-MCS-Luc2 tumor primary cells from mice in the Ctrl and Stress groups treated with *A. muciniphila* or saline. Spheres were counted from 24 replicate wells. (**t**) Mice were subjected to behavioral tests after completing the chronic stress paradigm. Time spent in the center was analyzed by the open field test (n = 8 mice per group). (**u**) Number of entries in open arms was analyzed using the elevated plus maze (n = 8 mice per group). (**v**) Body weight changes between Ctrl, Stress, Stress+AKK-Sup and Stress+Dead-AKK (n = 8 mice per group). Results are represented as mean ± s.d. (**a-d**, **f-h**, **l-n**, **q**, **r**, **t-v**) by one-way ANOVA, (**j, p**) by a two-tailed, unpaired Student’s *t*-test at the ethical end point, (**s**) by the likelihood ratio test. *P* values are as indicated.



**Fig. S4. *A. muciniphila* increases abundance of butyrate-producing bacteria to elevate butyrate levels.** (**a**) Levels of serum SCFAs of acetic acid (AA), propionic acid (PA), butyric acid (BA), isobutyric acid (IBA), valeric acid (VA), isovaleric acid (IVA), 4-methylvaleric acid (4-MVA), hexanoic acid (HA) in Ctrl and Stress groups inoculated with Py8119-pLVX-MCS-Luc2 or Py8119 cells (n = 10 mice per group). (**b-d**) Correlation of the gut microbiota with levels of fecal SCFAs (**b**), tumor SCFAs (**c**)**,** and serum SCFAs (**d**) in mice. (**e**) Levels of SCFAs of acetic acid (AA), propionic acid (PA), butyric acid (BA) in blank medium and AKK-supernatant groups (n = 6 biological replicates). (**f**) Schematic of the C57BL/6J mice inoculated with Py8119 cells with AKK administration. All mice were stopped after 21 days of Abx treatment before tumor implantation and then randomly divided into Ctrl and AKK groups. The Ctrl and AKK groups were treated with 200 μl (3 × 10^8^ CFU) *A. muciniphila* or saline per mice for 21 days (n = 6 mice per group). (**g**) Representative tumor image of Py8119 orthotopic tumors in the Abx-Ctrl and Abx+AKK groups (n = 6 mice per group). (**h**) Tumor volumes from Abx-Ctrl and Abx+AKK groups (n = 6 mice per group). (**i**) Levels of fecal butyric acid in mice inoculated with Py8119 tumors undergoing AKK or saline treatment (n = 12 biological replicates). (**j**) Heatmap of the selected most differentially abundant features at the species level in feces between Ctrl, Stress, AKK, Stress+AKK mice inoculated with Py8119 cells (n = 8 mice per group). Results are presented as mean ± s.d. (**a**, **e**, **h**, **i**) by two-tailed, unpaired Student’s *t*-test, (**b-d**) by Spearman’s rank test. For multiple comparisons, p-values were adjusted using the FDR correction. *P* values are as indicated.



**Fig. S5. Butyrate administration reverses psychological stress-triggered anxiety-like behavior and cancer stemness.** (**a**) Schematic of the C57BL/6J mice inoculated with Py8119 cells with sodium butyrate (NaBu) administration. The NaBu treatment groups intragastrically supplemented with 50 mg/kg, 100 mg/kg or 200 mg/kg NaBu in 200 μl (n = 6 mice per group) for 21 days. (**b**) Representative tumor image from C57BL/6J mice treated with dose of NaBu or saline (n = 6 mice per group). (**c**) Tumor volumes curves in mice treated with dose of NaBu or saline (n = 6 mice per group). (**d**) Body weight changes in the Ctrl and Stress groups treated with NaBu or saline (n = 6 mice per group). (**e**) Relative *Nanog* and *Sox2* mRNA expression in spontaneous tumors (n = 3 biological replicates). (**f**) Mice were subjected to behavioral tests after completing the chronic stress paradigm. Time spent in center were analyzed by the open field test (n = 8 mice per group). (**g**) Number of entries in open arms was analyzed using an elevated plus maze (n = 8 mice per group). (**h**) Body weight changes in Ctrl and Stress groups treated with NaBu or saline (n = 8 mice per group). (**i**) Relative *Nanog* and *Sox2* mRNA expression in Py8119-pLVX-MCS-Luc2 orthotopic tumors (n = 3 biological replicates). (**j**) ELDA were performed in Py8119-pLVX-MCS-Luc2 tumor primary cells. Stemness frequency with the upper and lower 95% confidence intervals indicate the frequency of one stem cell in the tumors. Spheres were counted from 24 replicate wells. (**k**) Representative images of spheroids formed by a single primary Py8119-pLVX-MCS-Luc2 tumor cell (left). Data represent the number of spheres per 300 cells (d > 50 μm) (middle) and the diameter of spheres (right) (Scale bars, 100 μm). (**l**) Mice were subjected to behavioral tests after completing the chronic stress paradigm. Time spent in center was analyzed by the open field test (n = 8 mice per group). (**m**) Number of entries in open arms was analyzed by the elevated plus maze (n = 8 mice per group). (**n**) Body weight changes in Ctrl and Stress mice treated with high fiber diet (HFD) or normal diet (n = 8 mice per group). Results are shown as the mean ± s.d. *P* values are as indicated. (**o**) Heatmap of the selected most differentially abundant features at the species level in feces between Ctrl, Stress, HFD, Stress+HFD mice inoculated with Py8119 cells (n = 8 mice per group). (**p**) Levels of fecal butyric acid in the Ctrl, Stress, HFD and Stress+HFD groups (n = 8 mice per group). (**q**) Relative mRNA expression of stemness genes in tumors was determined by RT-qPCR (n = 3 biological replicates). Results are presented as mean ± s.d. (**c**) by two-tailed, unpaired Student’s *t*-test at the ethical end point, (**d-i**, **k-n**, **p**, **q**) by one-way ANOVA, (**j**) by the likelihood ratio test. For multiple comparisons, p-values were adjusted using the FDR correction. *P* values are as indicated.



**Fig. S6. LRP5 mediates butyrate-blocked β-catenin signaling and cancer stemness**. (**a**) Relative mRNA level of *LRP5*, *NANOG* and *SOX2* was determined between MDA- MB-231 non-sphere and spherical cells (n = 3 biological replicates). (**b**) SK-BR-3 cells and MDA-MB-231 cells were incubated with different concentrations of NaBu for 48 hours. Cell viabilities were measured by CCK8 assay (n = 6 biological replicates). (**c**) Relative expression levels of *LRP5* mRNA in MDA-MB-231 cells treated with NaBu for 48h in dose-dependent manner (n = 3 biological replicates). (**d-e**) Relative *Lrp5* mRNA expression (**d**) and relative LRP5 and β-catenin protein levels (**e**) in Py8119-pLVX-MCS-Luc2 tumor primary cells of SPF and GF mice following the Ctrl and Stress treatments (n = 3 biological replicates). (**f-g**) Relative *Lrp5* mRNA expression (**f**) and relative LRP5 and β-catenin protein levels (**g**) of spontaneous tumors in the Ctrl and Stress groups treated with Abx or H_2_O (n = 3 biological replicates). (**h-i**) Relative *LRP5* mRNA expression (**h**) and relative LRP5 and β-catenin protein levels (**i**) of primary MDA-MB-231 tumors in the Ctrl and Stress groups treated with Abx or H_2_O (n = 3 biological replicates). (**j**) Relative expression of the indicated genes in the Ctrl and LRP5-forced expression MDA-MB-231 cells treated with NaBu (4 mM) for 48h or vehicle (n = 3 biological replicates). (**k**) Relative expression of the indicated genes in the Ctrl and MCM5-forced expression SK-BR-3 cells treated with NaBu (4 mM) for 48h or vehicle (n = 3 biological replicates). (**l**) ALDH^+^ cells in the Ctrl and LRP5-forced expression MDA-MB-231 cells treated with NaBu (4 mM) for 48h or vehicle (n = 3 biological replicates). (**m**) ALDH^+^ cells in the Ctrl and MCM5-forced expression SK-BR-3 cells treated with NaBu (4 mM) for 48h or vehicle (n = 3 biological replicates). (**n**) ELDA were performed in the 4 groups and representative sphere images are shown (Scale bars, 50 μm). Stemness frequency with the upper and lower 95% confidence intervals showing the frequency of one stem cell in the tumors. Spheres were counted from 24 replicate wells. (**o**) Representative images of spheroids formed by single cells in the 4 groups (left), distribution patterns of the number of spheres per 200 cells (d > 50 μm) (middle) and sphere diameter (right) (Scale bars, 100 μm). (**p**) Relative LRP5, p-GSK3β, GSK3β, and β-catenin protein levels in Ctrl and LRP5-forced expression MDA-MB-231 cells treated with NaBu (4 mM) for 48h or vehicle. (**q**) Representative images of β-catenin protein immunofluorescence (green) was in Ctrl and LRP5-forced expression MDA-MB-231 cells treated with NaBu (4 mM) for 48h or vehicle. The nucleus was stained with DAPI (blue) (Scale bars, 25 μm). (**r**) Western blots showing the Expression of nuclear and cytoplasmic protein in extracts from the Ctrl and LRP5-forced expression MDA-MB-231 cells treated with NaBu (4 mM) for 48h or vehicle. Actin served as the cytoplasmic internal loading control and Lamin B1 as the nuclear internal control. Results are presented as mean ± s.d. (**a-c**) by two-tailed, unpaired Student’s *t*-test, (**d**, **f**, **h**, **j-m**, **o**) by one-way ANOVA, (**n**) by the likelihood ratio test. *P* values are as indicated.



**Fig. S7. Butyrate elevates ZFP36 to promote LRP5 mRNA decay, inhibiting β-catenin activation and cancer stemness.** (**a-b**) Relative pre-*LRP5* mRNA expression of SK-BR-3 cells (**a**) and MDA-MB-231 cells (**b**) treated with NaBu for 48h in a dose-dependent manner (n = 3 biological replicates). (**c**) Relative *ZFP36* and pre-*ZFP36* mRNA levels in BT549 cells treated with NaBu for 48h in a dose-dependent manner (n = 3 biological replicates). (**d-e**) Relative *ZFP36L1* and *ZFP36L2* mRNA in SK-BR-3 cells (**d**) and MDA-MB-231 cells (**e**) treated with NaBu for 48h in a dose-dependent manner (n = 3 biological replicates). (**f**) Relative ZFP36 protein levels of SK-BR-3 and MDA-MB-231 cells treated with NaBu for 48h in dose-dependent manner. (**g**) Expression of *FFAR2*, *FFAR3*, and *HCAR2* in SK-BR-3 and MDA-MB-231 cells after transfection with the siRNA targeting these three GPRs to test for successful knockdown (n = 3 biological replicates). (**h**) Relative ZFP36, LRP5, β-catenin, Histone H3-K9, and Histone H3 protein levels in MDA-MB-231 cells treated with NaBu (4 mM) and TSA (1 μM). (**i-j**) Relative *ZFP36* and *LRP5* mRNA expression (**i**) and stability of *LRP5* mRNA (**j**) in MDA-MB-231 cells with ZFP36 knocked down and treated with 4 mM NaBu (n = 3 biological replicates). (**k**) Relative ZFP36, LRP5, p-GSK3β, GSK3β, and β-catenin protein levels in MDA-MB-231 cells with ZFP36 knocked down and treated with NaBu (4 mM). (**l**) Representative immunofluorescence images of β-catenin protein (green) in MDA-MB-231 cells with ZFP36 knocked down and treated with NaBu (4 mM). Nuclei were identified using DAPI (blue) (Scale bars, 25 μm). (**m-n**) Relative *NANOG* and *SOX2* levels in SK-BR-3 cells (**m**) and MDA-MB-231 cells (**n**) with ZFP36 knocked down and treated with 4 mM NaBu (n = 3 biological replicates). (**o**) ALDH^+^ cells in MDA-MB-231 cells with ZFP36 knocked down and treated with NaBu (4 mM) (n = 3 biological replicates). Results are presented as mean ± s.d. (**a-e**, **g**) by two-tailed, unpaired Student’s *t*-test, (**i**, **j**, **m**-**o**) by one-way ANOVA. *P* values are as indicated.



**Fig. S8. Clinical relevance of HADS, *A. muciniphila*, butyrate and molecular markers in breast cancer patients**. (**a-b**) Pearson correlation between HADS scores and *A. muciniphila* abundance (**a**) and butyric acid (**b**) of feces in cohort 1 breast cancer patients (n = 57). (**c**) Pearson correlation between HADS scores and butyric acid of serum in cohort 1 breast cancer patients (n = 57). (**d**-**e**) Pearson correlation between *A. muciniphila* abundance with butyric acid level in feces (**d**) and butyric acid of serum (**e**) in cohort 1 breast cancer patients (n = 57). (**f**) Log2-transformed relative *Akkermansia muciniphila* expression in feces of cohort 2 breast cancer patients between HADS low (n = 24) and high (n = 34) patients. (**g**) Butyric acid level in feces of cohort 2 breast cancer patients between HADS low (n = 24) and high (n = 34) patients. (**h-i**) Pearson correlation between HADS scores with log2 relative *A. muciniphila* expression (**h**) and butyric acid (**i**) of feces in cohort 2 breast cancer patients (n = 58). (**j**) Pearson correlation between log2 relative *A. muciniphila* expression with butyric acid level in feces in cohort 2 breast cancer patients (n = 58). (**k-n**) Pearson correlation between HADS scores and ZFP36 (**k**), LRP5 (**l**), β-catenin (**m**) and NANOG (**n**) expression in cohort 1 breast cancer patients (n = 57). (**o-r**) Pearson correlation between *A. muciniphila* abundance and ZFP36 (**o**), LRP5 (**p**), β-catenin (**q**) and NANOG (**r**) expression in cohort 1 breast cancer patients (n = 57). (**s**) Pearson correlation between ZFP36 score and LRP5 score of tumors in cohort 1 breast cancer patients (n = 57). (**t**) Pearson correlation between LRP5 score and β-catenin score of tumors in cohort 1 breast cancer patients (n = 57). (**u**) Pearson correlation between ZFP36 score and β-catenin score of tumors in cohort 1 breast cancer patients (n = 57). (**v**) Pearson correlation between LRP5 score and NANOG score of tumors in cohort 1 breast cancer patients (n = 57). (**w**-**y**) Relative *NANOG* (**w**)*, SOX2* (**x**), and *Ki67* (**y**) mRNA expression between butyric acid in serum low (n = 19) and high (n = 20) groups in cohort 1 breast cancer patients. Results are presented as mean ± s.d. (**a-e**, **h-v**) by Pearson’s rank test, (**f**, **g**, **w**-**y**) by two-tailed, unpaired Student’s *t*-test. *P* values are as indicated.

Table S1. Clinical information of cohort 1 (57 breast cancer patients) and cohort 2 (58 breast cancer patients)

| **Cohort 1** Overall (n=57) | | **Cohort 2** Overall (n=58) | |
| --- | --- | --- | --- |
| Factor | NO. | Factor | NO. |
| **Sex** |  | **Sex** |  |
| Female | 57 | Female | 57 |
| Male | - | Male | - |
| **Age**, years |  | **Age**, years |  |
| ≤ 54 | 30 | ≤ 54 | 32 |
| > 54 | 27 | > 54 | 26 |
| **HADS score** |  | **HADS score** |  |
| ≤ 7 | 24 | ≤ 7 | 24 |
| > 7 | 33 | > 7 | 34 |
| ***A. muciniphila* abundance**  (Metagenomics data) |  | ***A. muciniphila* abundance**  (log2 qPCR data) |  |
| ≤ 23.54 | 29 | ≤ 18.70 | 29 |
| > 23.54 | 28 | > 18.70 | 29 |
| **Butyric acid level in fecal**  (μg/g) |  | **Butyric acid level in fecal**  (μmol/mg) |  |
| ≤ 917 | 29 | ≤ 42.17 | 29 |
| > 917 | 28 | > 42.17 | 29 |
| **Butyric acid level in serum**  (μM) |  | - |  |
| ≤ 16.52 | 29 | **-** | - |
| > 16.52 | 28 | **-** | - |
| **Start year** |  | **Start year** |  |
| 2022 | 37 | 2024 | 58 |
| 2023 | 20 |  |  |

**Table S2. Correlation between HADS and clinicopathological parameters**

| Factor | HADS low  NO. % | | HADS high  NO. % | | *P* value |
| --- | --- | --- | --- | --- | --- |
| All patients | 24 | 42.1 | 33 | 57.9 |  |
| **Age**, years |  |  |  |  | 0.60 |
| ≤ 54 | 14 | 46.7 | 16 | 53.3 |  |
| > 54 | 10 | 37 | 17 | 63 |  |
| **Tumor size**, cm |  |  |  |  | 0.2 |
| ≤ 1 | 15 | 51.7 | 14 | 48.3 |  |
| > 1 | 9 | 32.1 | 19 | 67.9 |  |
| **Node status** |  |  |  |  | 0.005 |
| Negative | 13 | 72.2 | 5 | 27.8 |  |
| Positive | 11 | 28.2 | 28 | 71.8 |  |
| **Distant metastasis** |  |  |  |  | 0.01 |
| Negative | 19 | 57.6 | 14 | 42.4 |  |
| Positive | 5 | 20.8 | 19 | 79.2 |  |
| **Stage** |  |  |  |  | 0.01 |
| Ⅰ or Ⅱ | 19 | 57.6 | 14 | 42.4 |  |
| Ⅲ | 5 | 20.8 | 19 | 79.2 |  |
| **ER status** |  |  |  |  | 1 |
| Negative | 4 | 40 | 6 | 60 |  |
| Positive | 20 | 42.6 | 27 | 57.4 |  |
| **PR status** |  |  |  |  | 0.3 |
| Negative | 6 | 54.5 | 5 | 45.5 |  |
| Positive | 17 | 37.8 | 28 | 62.2 |  |
| Missing | 1 |  |  |  |  |
| **HER2 status** |  |  |  |  | 0.52 |
| Negative | 2 | 28.6 | 5 | 71.4 |  |
| Positive | 21 | 43.75 | 27 | 56.25 |  |
| Missing | 1 |  | 1 |  |  |
| **Ki67 status** |  |  |  |  | 0.4 |
| ≤ 0.3 | 13 | 46.4 | 15 | 53.6 |  |
| > 0.3 | 6 | 30 | 14 | 70 |  |
| Missing | 5 |  | 4 |  |  |
| **CA153**, U/mL |  |  |  |  | 0.005 |
| ≤ 9.48 | 18 | 62.1 | 11 | 37.9 |  |
| > 9.48 | 6 | 21.4 | 22 | 78.6 |  |
| **Epinephrine**, nmol/L |  |  |  |  | 0.3 |
| ≤ 0.19 | 16 | 50 | 16 | 50 |  |
| > 0.19 | 8 | 32 | 17 | 68 |  |
| **Norepinephrine,** nmol/L |  |  |  |  | 0.02 |
| ≤ 0.43 | 18 | 58.1 | 13 | 41.9 |  |
| > 0.43 | 6 | 23.1 | 20 | 776.9 |  |
| **Estradiol**, μg/L |  |  |  |  | 0.02 |
| ≤ 20.99 | 17 | 58.6 | 12 | 41.4 |  |
| > 20.99 | 7 | 25 | 21 | 75 |  |
| **Thyroid hormone**, pmol/L |  |  |  |  | 0.005 |
| ≤ 15.64 | 18 | 62.1 | 11 | 37.9 |  |
| > 15.64 | 6 | 21.4 | 22 | 78.6 |  |
| **Cortisol**, pg/ml |  |  |  |  | 0.02 |
| ≤ 651.71 | 17 | 58.6 | 12 | 41.4 |  |
| > 651.71 | 7 | 25 | 21 | 75 |  |

Statistical significance was determined by Pearson’s Chi-squared (*χ^2^*) test and Fisher's Exact Test (ER status, PR status and HER2 status).

Table S3. List of primer sequence for plasmid construction

| **Oligonucleotides (Sangon Biotech)** | | |
| --- | --- | --- |
| **Plasmids** | **Sense (5’-3’)** | **Antisense (5’-3’)** |
| PCDH-LRP5 | GCTCTAGAGCGCCACCATGGAGGCAGCGCCGCCCGGGCCGC | CGGAATTCCGTCAGGATGAG  TCCGTGCAGGGGGACGGA |
| PCDH-MCM5 | GCTCTAGAGCGCCACCATGTCGGGATTCGACGATCCTGGCA | CGGAATTCCGTCACTTGAGGCGGTAGAGAACCTTG |
| **Luciferase constructs** | **Sense (5’-3’)** | **Antisense (5’-3’)** |
| psiCHECK2-LRP5-3’UTR-WT | CCCTCGAGGGTCCCCCTGCACGGACTCATCCTGA | TTGCGGCCGCAATTCTGTTT  TACAAAATTAAGTTTATA |
| psiCHECK2-LRP5-3’UTR-Mut1 | ATTTTATGACCCAAAAAATAAATATAATTGGGATTTTAAAAACATG | TTTATTTTTTGGGTCATAAA  ATATATTTTTTTCTTTGTTCATATTTA |
| psiCHECK2-LRP5-3’UTR-Mut2 | GGAGAAATACCCATAAACTTAATTTTGTAAAACAGAATTGCGGCCG | TAAGTTTATGGGTATTTCTC  CACTGTACAAAGTTCTCCC |

Table S4. List of siRNA sequence

| **Oligonucleotides** | |
| --- | --- |
| **siRNA (GenePharma)** | **sense（5' -3' ）** |
| siNC | UUCUCCGAACGUGUCACGU |
| siZFP36 | GGGACAGUAAUCAAGUAAU |
| siFFAR2 | CUACGAGAACUUCACCGAU |
| siFFAR3 | CGUGGUCUACGUCAUAGAA |
| siHCAR2 | GAGAUGACUUCAUUGUCAA |

| **Table S5. List of PCR amplification and sequencing primersOligonucleotides** | | |
| --- | --- | --- |
| **Fecal DNA qPCR primers**  **(Sangon Biotech)** | **Sense (5’-3’)** | **Antisense (5’-3’)** |
| *Firmicutes* | TGAAACTYAAAGGAATTGACG | ACCATGCACCACCTGTC |
| *Actinobacteria* | TACGGCCGCAAGGCTA | TCRTCCCCACCTTCCTCCG |
| *Akkermansia Muciniphila* | CAGCACGTGAAGGTGGGGAC | CCTTGCGGTTGGCTTCAGAT |
| *Eubacteria* (all bacteria) | ACTCCTACGGGAGGCAGCAGT | ATTACCGCGGCTGCTGGC |
| **RT-qPCR primers**  **(Sangon Biotech)** | **Sense (5’-3’)** | **Antisense (5’-3’)** |
| Mus-Actb | GAGGTATCCTGACCCTGAAGTA | CACACGCAGCTCATTGTAGA |
| Mus-Sox2 | GCGGAGTGGAAACTTTTGTCC | CGGGAAGCGTGTACTTATCCTT |
| Mus-Nanog | CACAGTTTGCCTAGTTCTGAGG | GCAAGAATAGTTCTCGGGATGAA |
| Mus-LRP5 | ACGTCCCGTAAGGTTCTCTTC | GCCAGTAAATGTCGGAGTCTAC |
| Hum-ACTB | TTGCCGACAGGATGCAGAAGGA | AGGTGGACAGCGAGGCCAGGAT |
| Hum-ZFP36 | GACTGAGCTATGTCGGACCTT | GAGTTCCGTCTTGTATTTGGGG |
| Hum-LRP5 | ACTCGCTGTGAGGAGGACAAT | GGCAGGCGCATGTGTAGAA |
| Hum-MCM5 | ATGTCGGGATTCGACGATCCT | CCAGGTTGTAATGCCGCTTG |
| Hum-pre-ZFP36 | ATCCGACTTCTGTCTCTCCAG | GTTTCACAACTGGAGAAACCG |
| Hum-ZFP36L1 | TCCAGCATAGCTTTAGCTTTGC | GGTCATCGGCGCTCAGAATAG |
| Hum-ZFP36L2 | CAACTCCACGCGCTACAAGA | CACTTTTCGCCGTACTTGCAC |
| Hum-pre-LRP5 | CCACAGCCTCGCCGCTCCTGCTA | GGCTCCCTTGGAAAACTGGAAGT |
| Hum-MCM5 | ATGTCGGGATTCGACGATCCT | CCAGGTTGTAATGCCGCTTG |
| Hum-FFAR2 | TGCTACGAGAACTTCACCGAT | GGAGAGCATGATCCACACAAAAC |
| Hum-FFAR3 | TTCACCACCATCTATCTCACCG | TGTGAGTGTTCACTGGTCTTTC |
| Hum-HCAR2 | ATGTTGGCTATGAACCGCCAG | GCTGCTGTCCGATTGGAGA |
| ChIP | CTGCCTGGAGGCGGAAGTG | GACTGGAGAGACAGAAGTCGGA |
| RIP | TCTGGCTTCTCTGTGCCCCTGTAA | CCAGCGGCCGCAATTCTGTTTTAC |
