# Supplementary material for: Gut dysbiosis conveys psychological stress to activate LRP5/β-catenin pathway promoting cancer stemness
Source: Signal Transduct Target Ther. 2025 Mar 5;10:79. doi: 10.1038/s41392-025-02159-1 (PMC11880501; doi:10.1038/s41392-025-02159-1)

Full unedited gel for Fig. 1

Fig. 1i

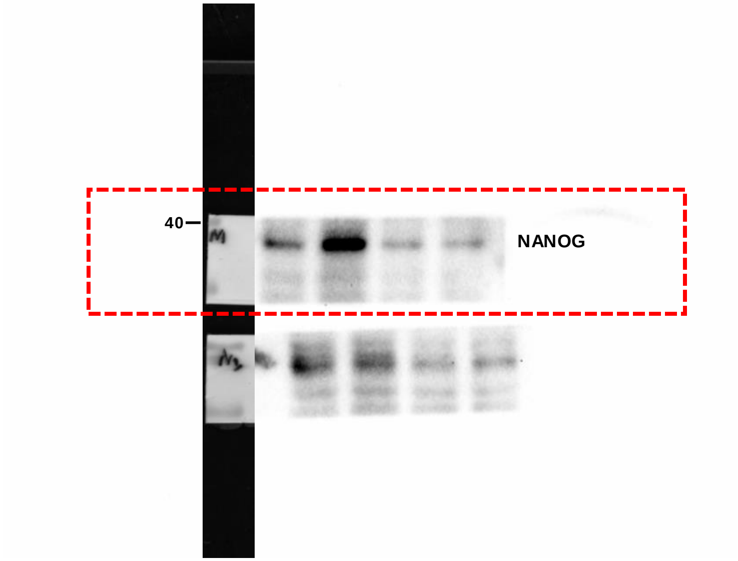

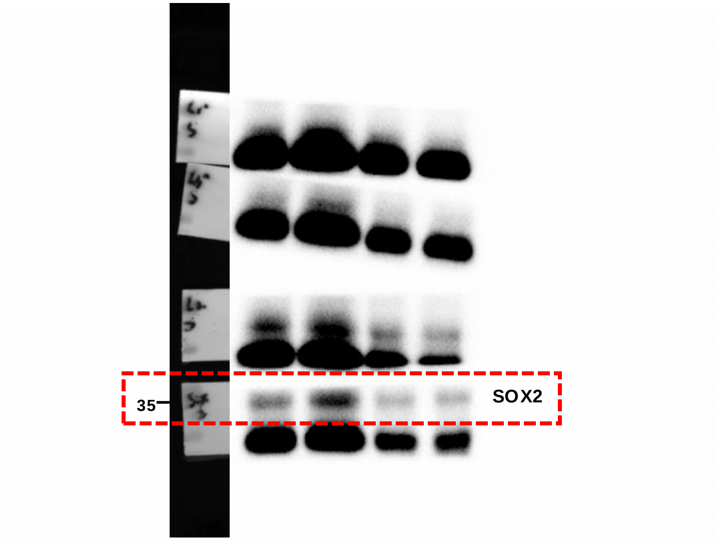

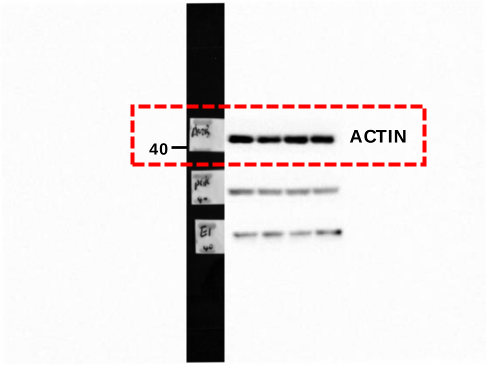

Full unedited gel for Fig. 3

Fig. 3i

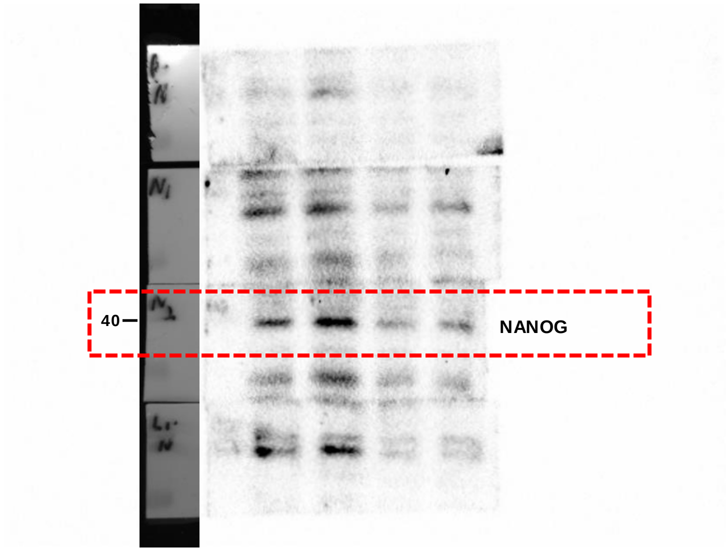

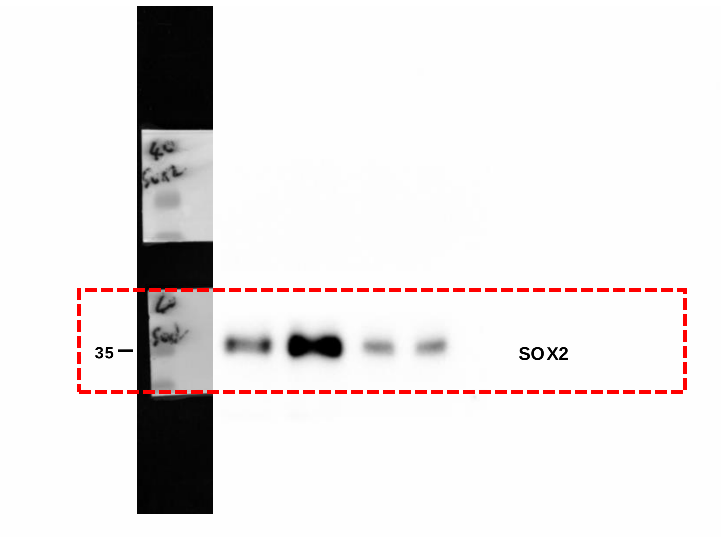

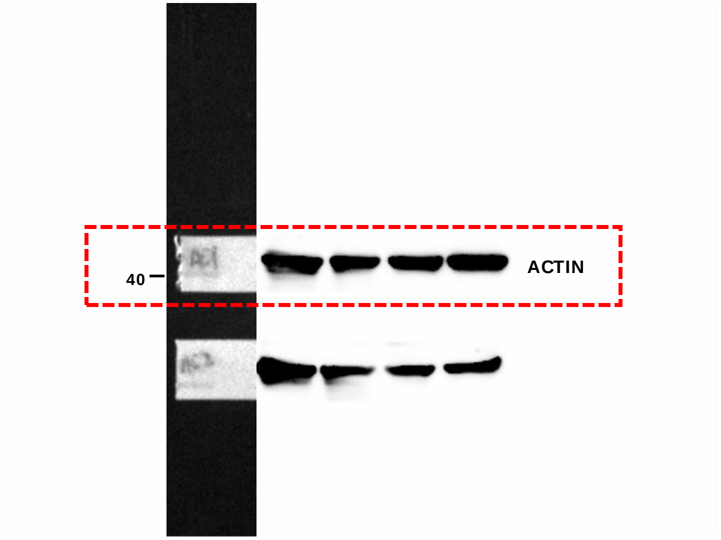

Full unedited gel for Fig. 4

Fig. 4j

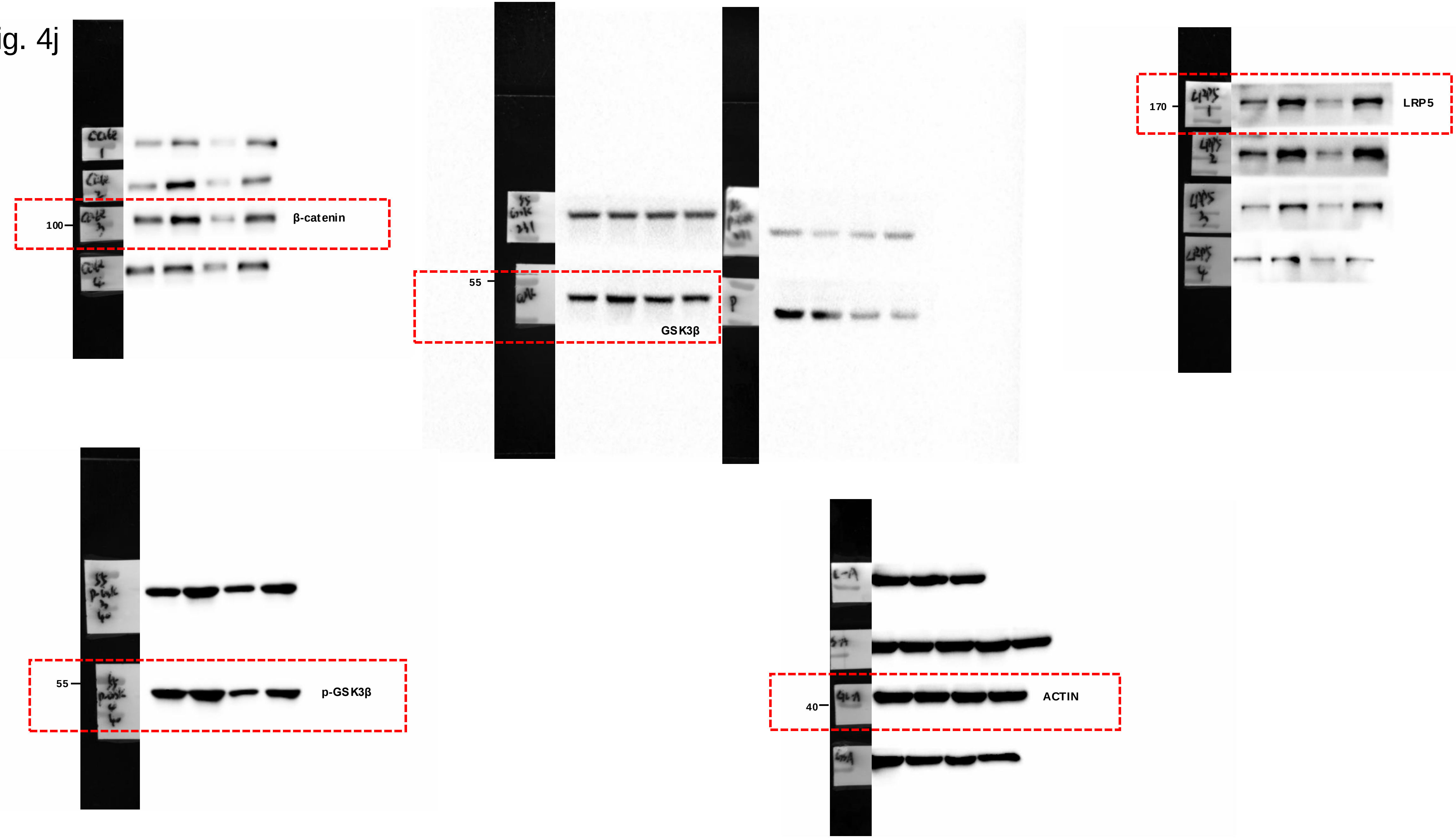

Full unedited gel for Fig. 4

Fig. 4l

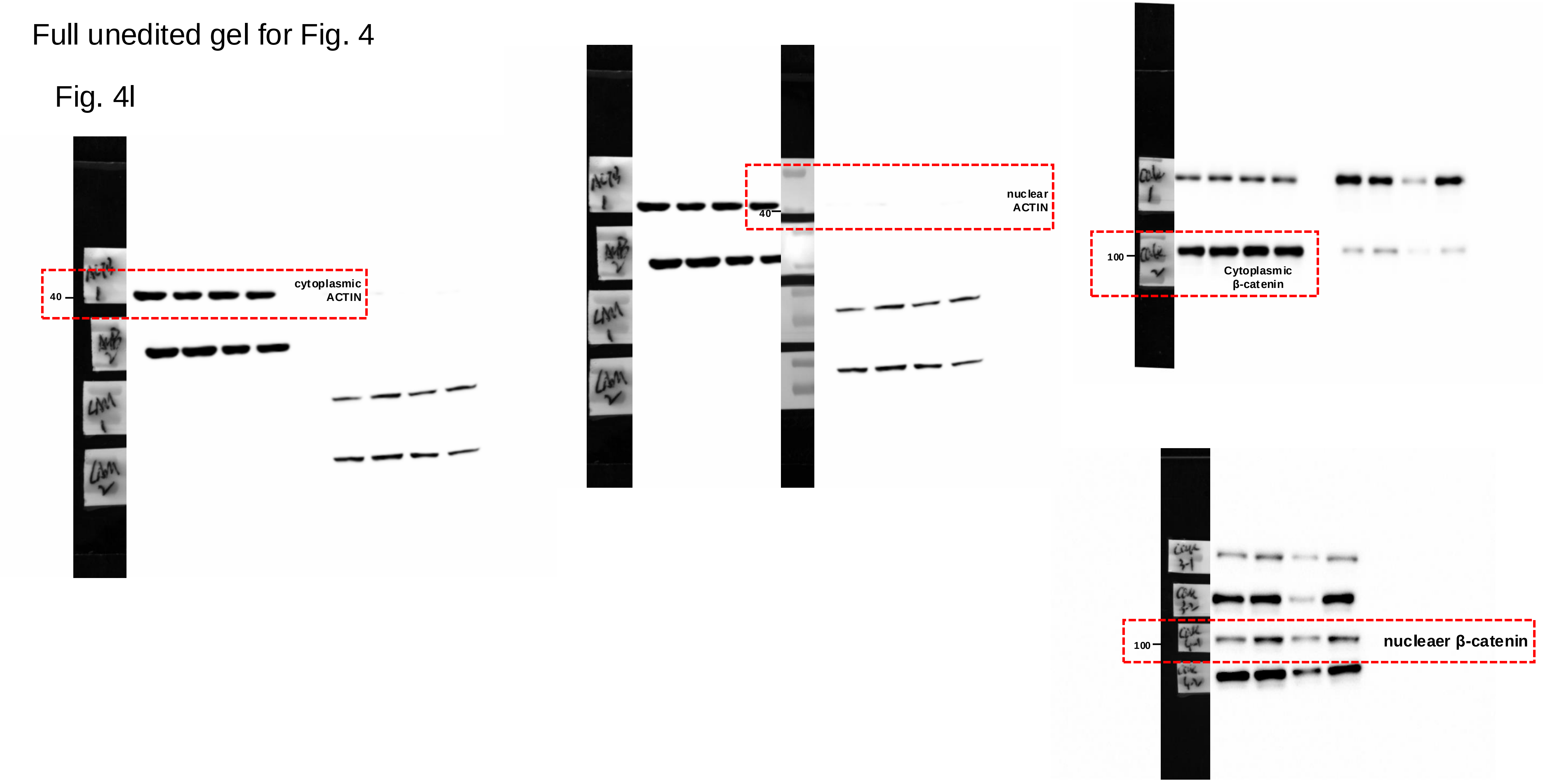

Full unedited gel for Fig. 4

Fig. 4l

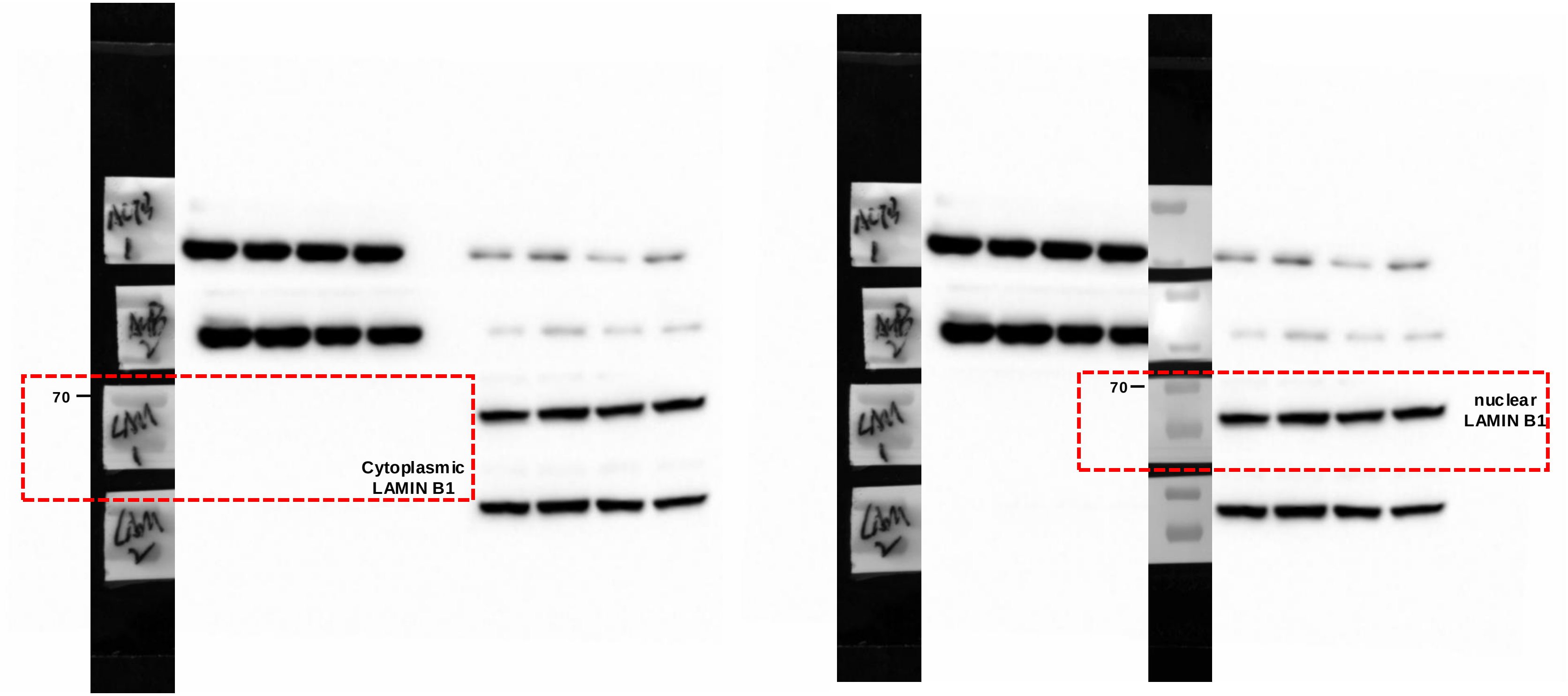

Full unedited gel for Fig. 5

Fig. 5f

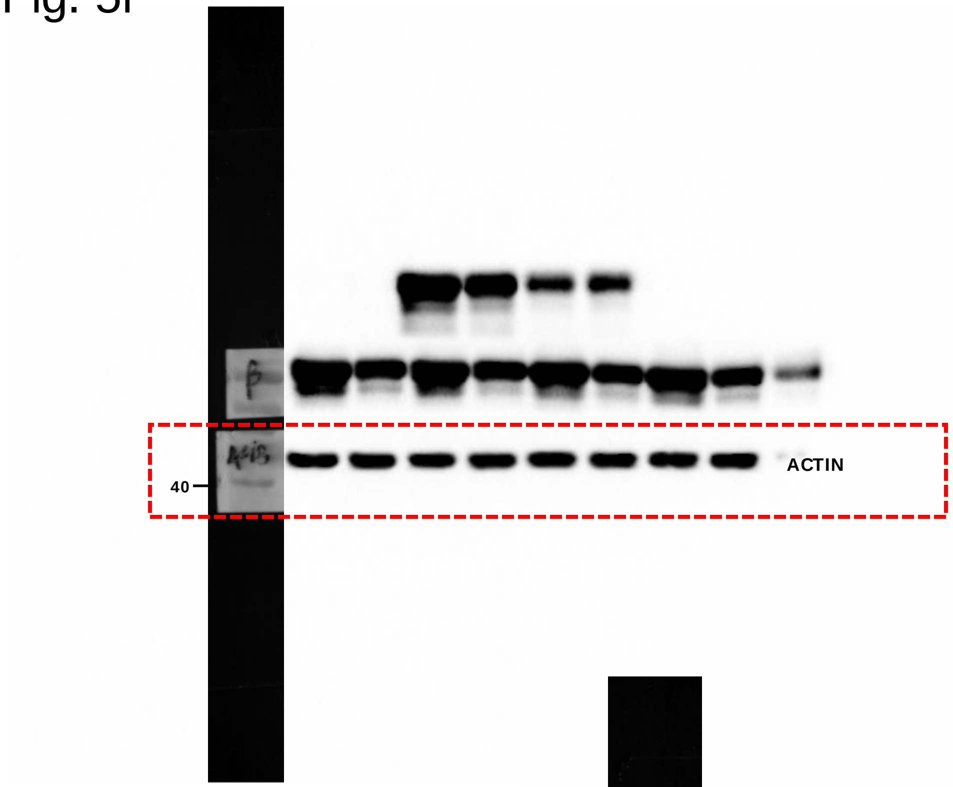

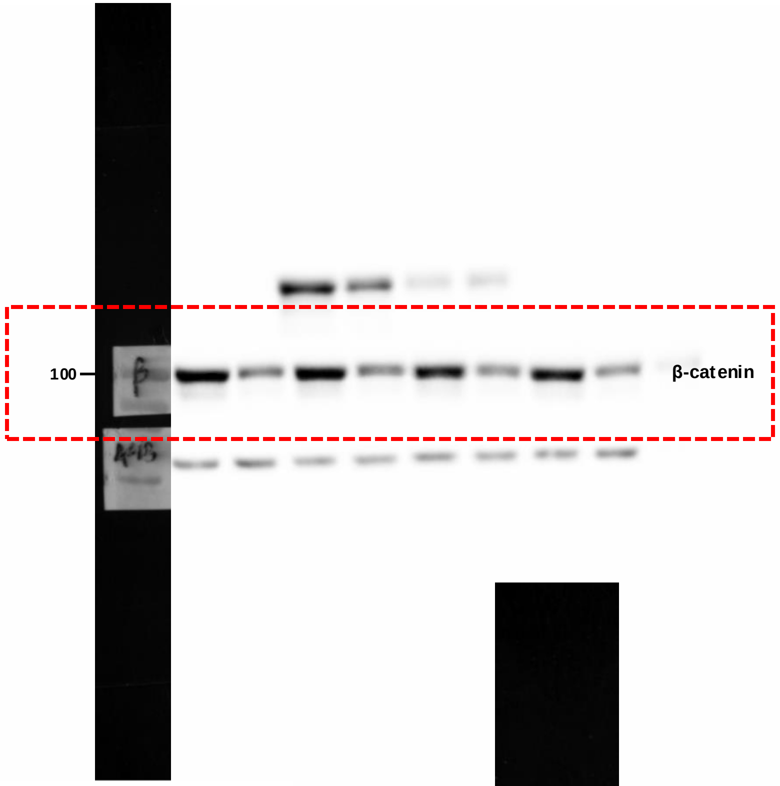

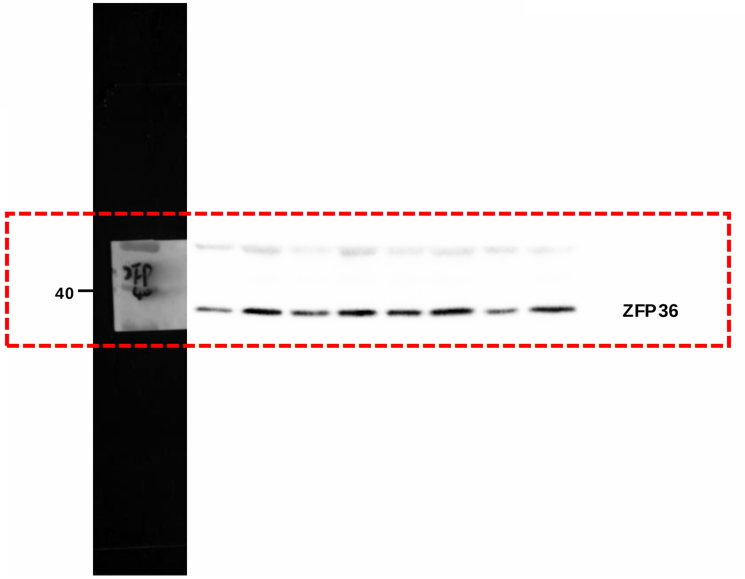

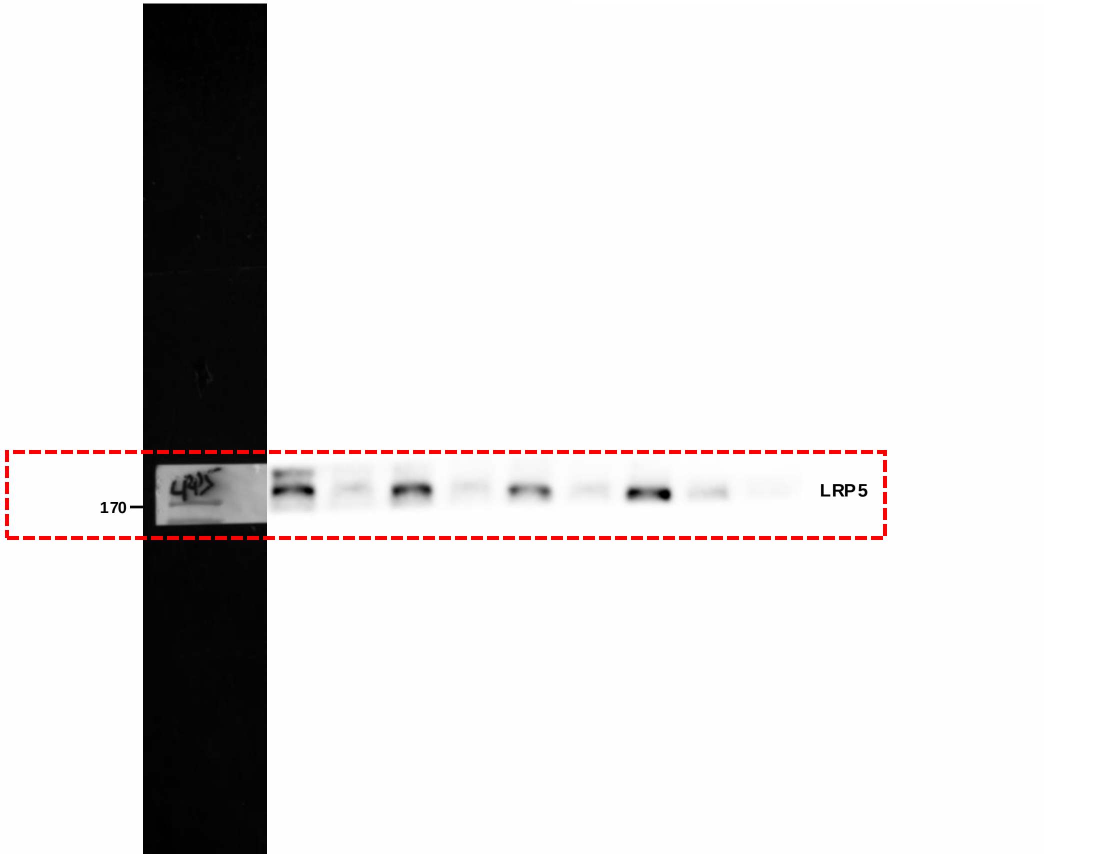

Full unedited gel for Fig. 5

Fig. 5g

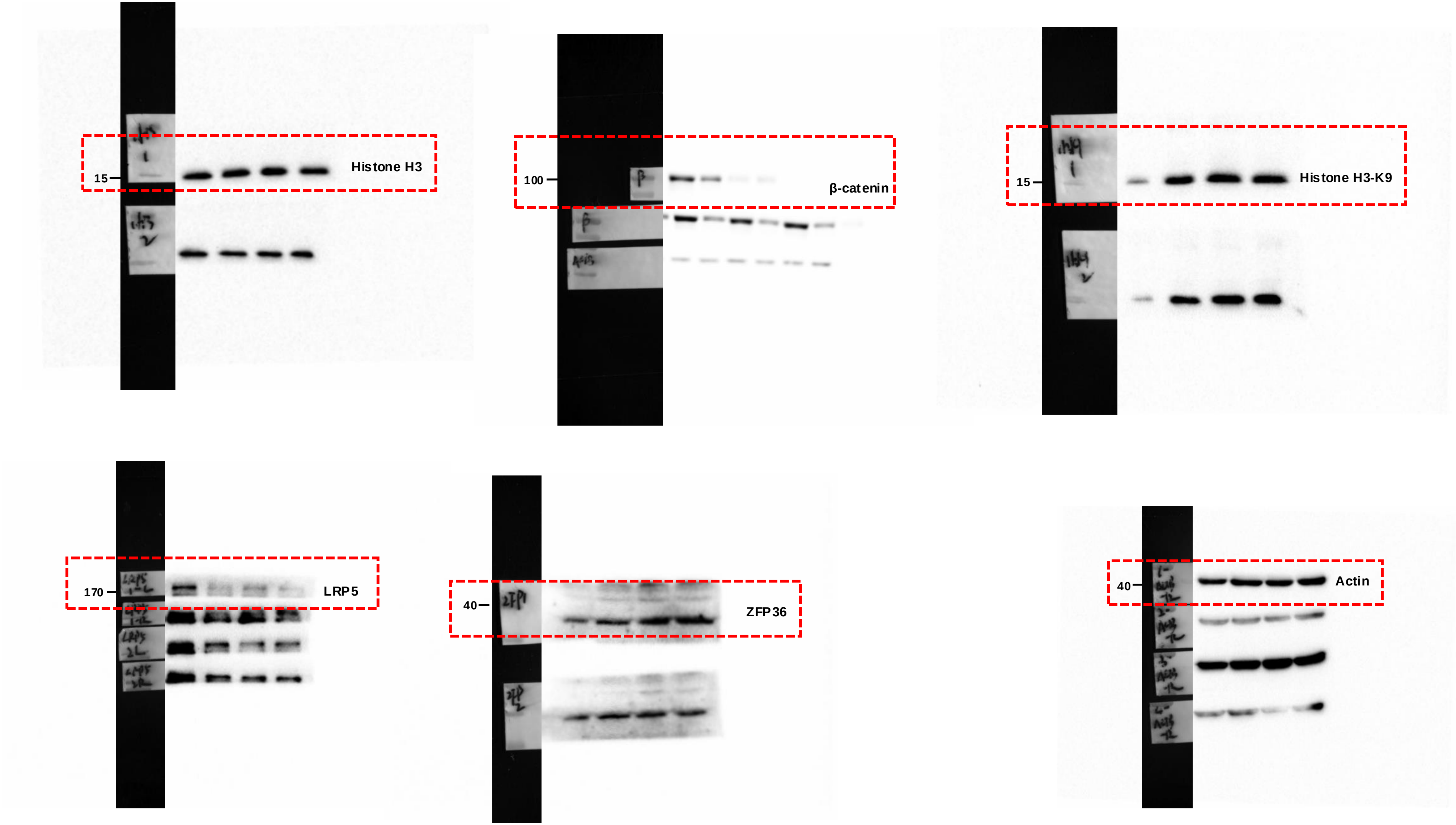

Full unedited gel for Fig. 5

Fig. 5n

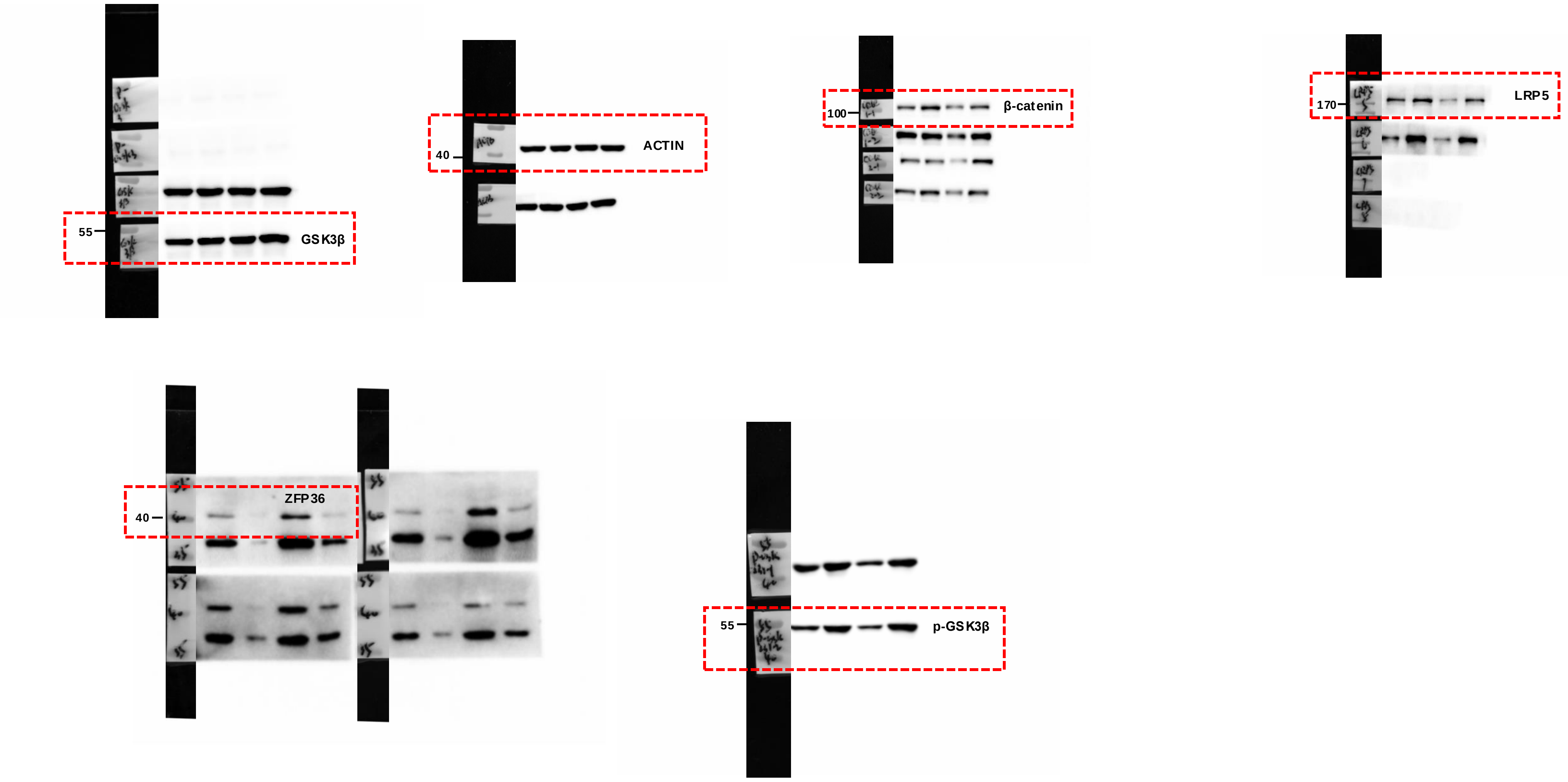

Full unedited gel for Fig. 6

Fig. 6m

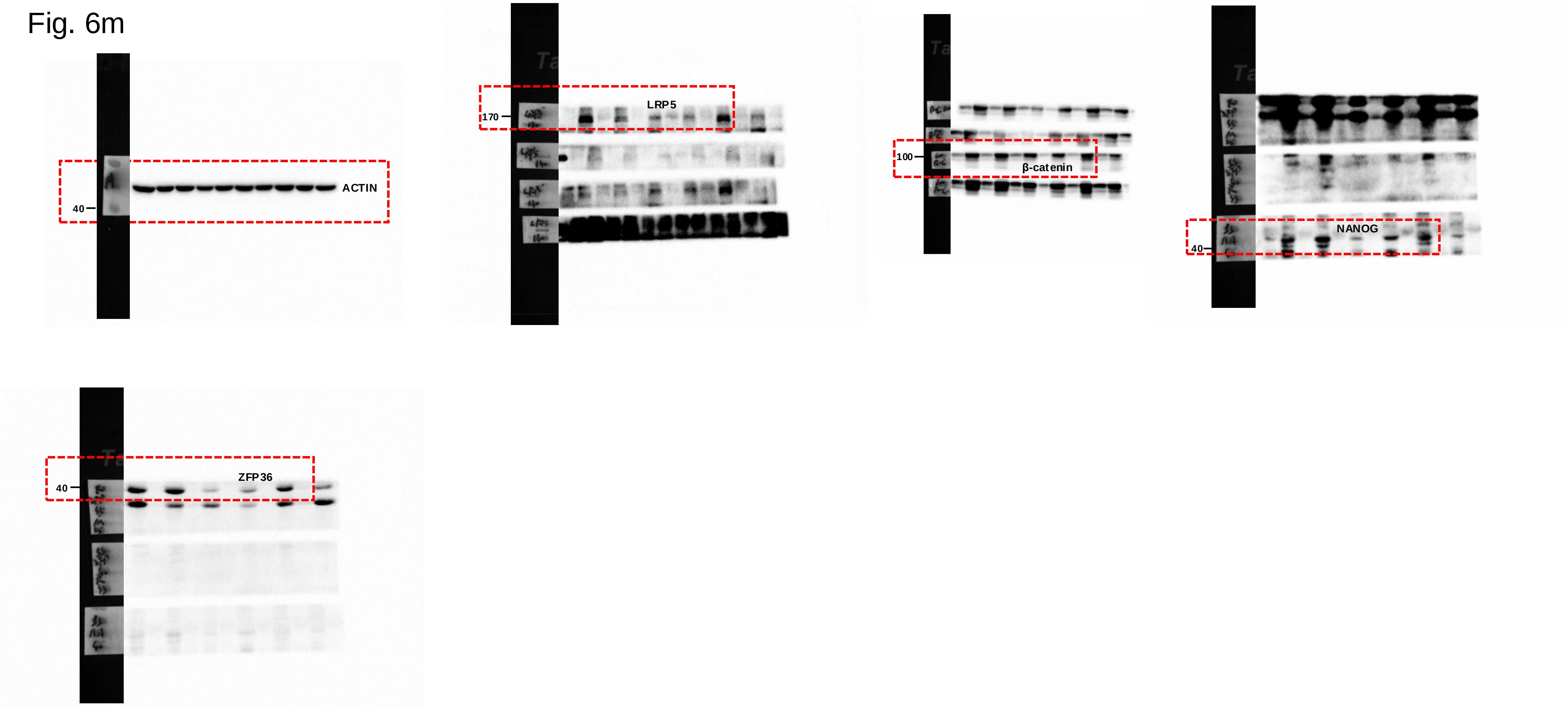

Full unedited gel for Fig. S6

Fig. S6e

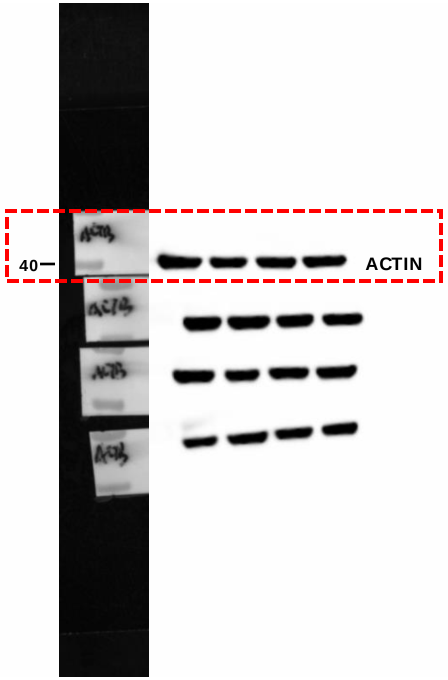

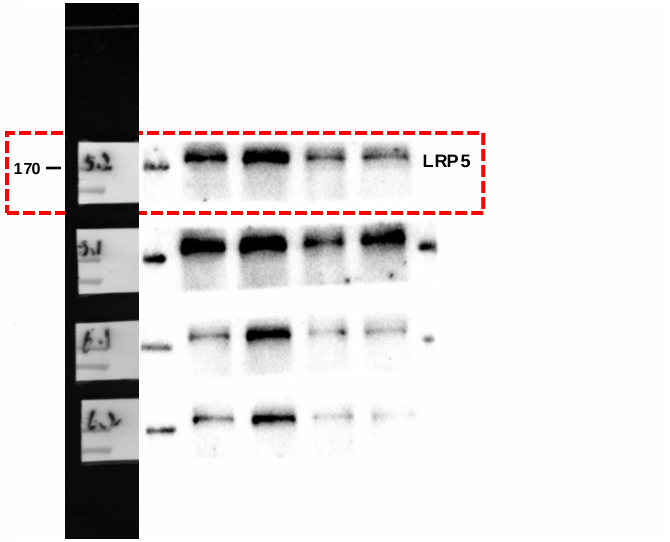

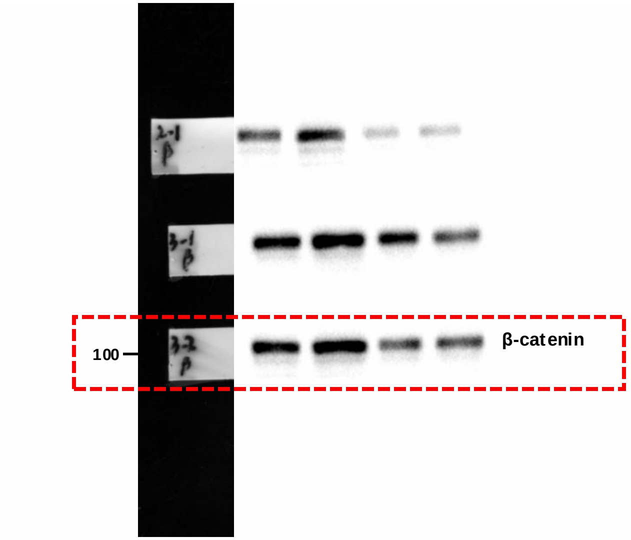

Full unedited gel for Fig. S6 Fig. S6g

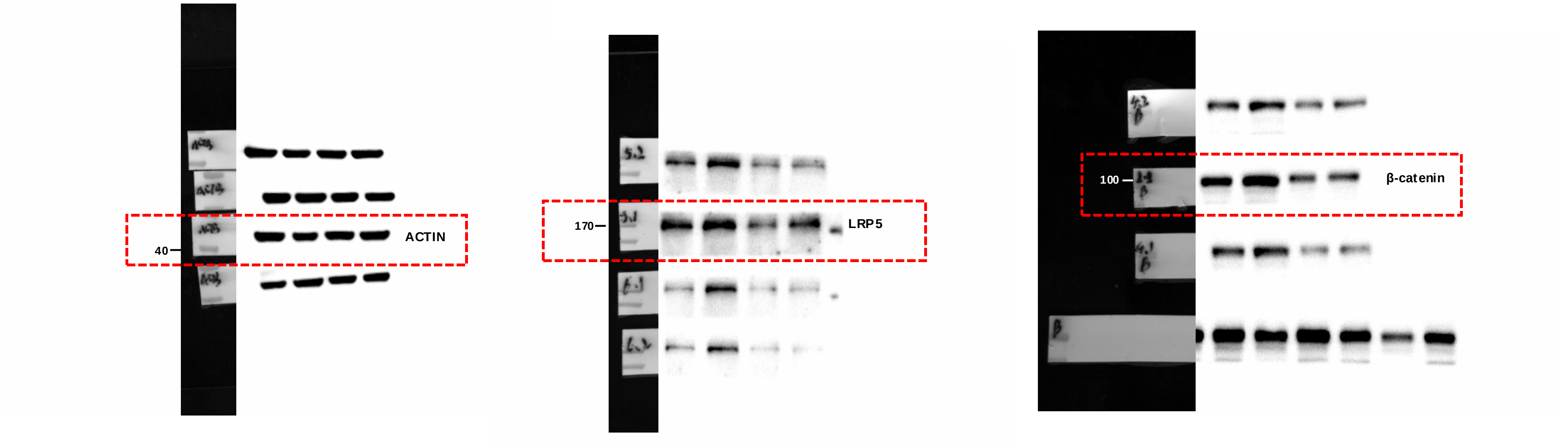

Full unedited gel for Fig. S6 Fig. S6i

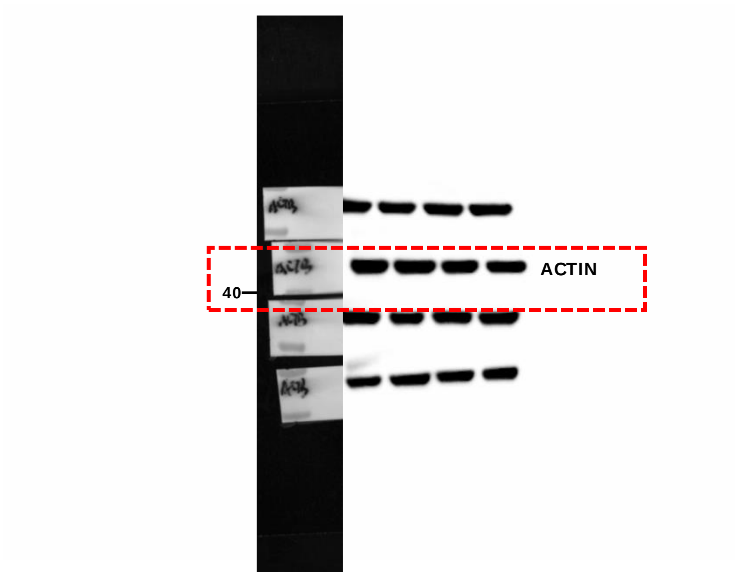

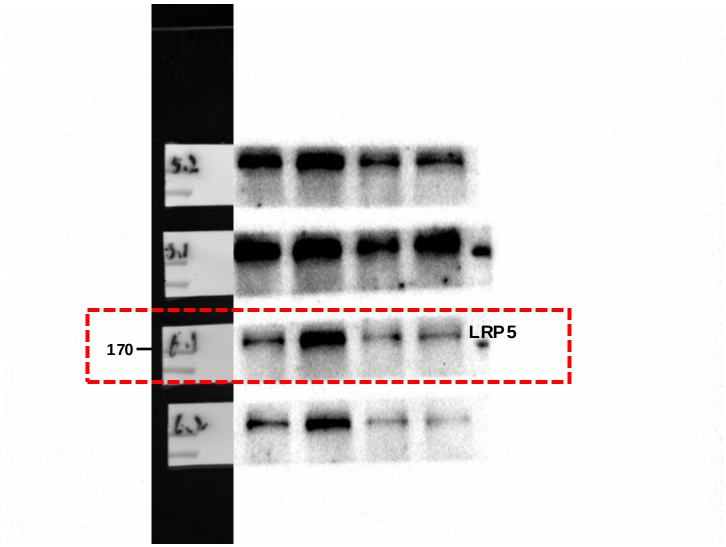

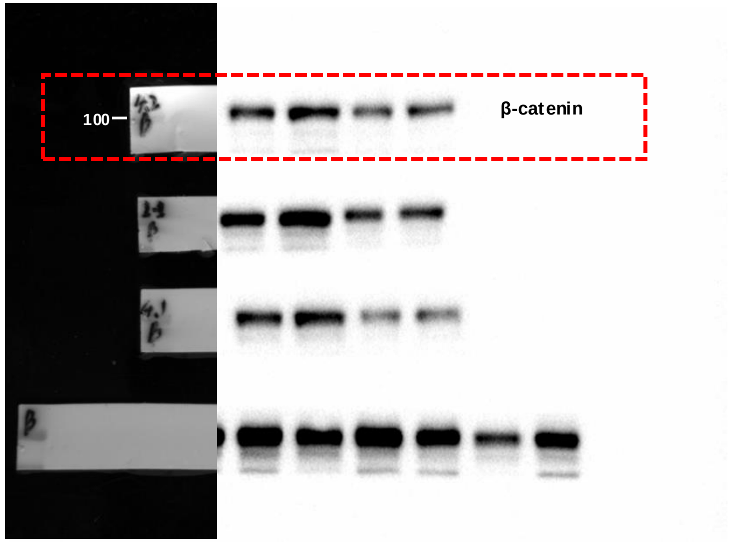

Full unedited gel for Fig. S6 Fig. S6p

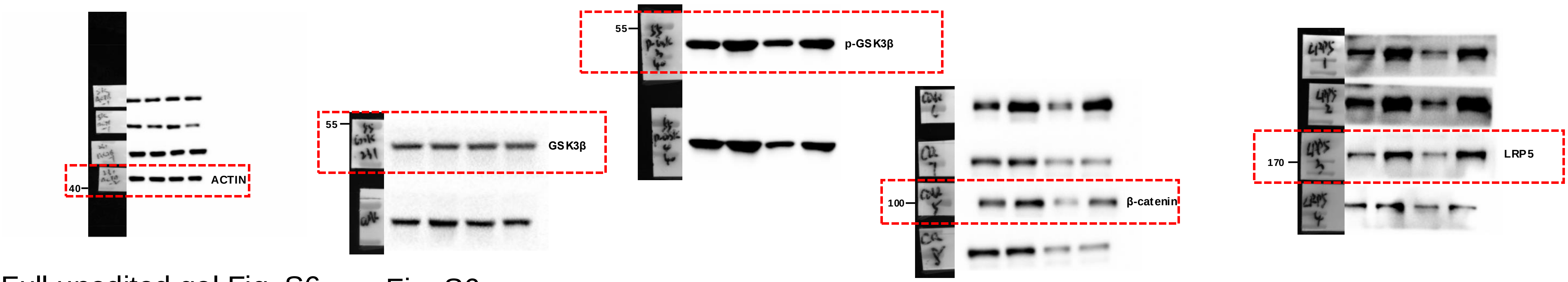

Full unedited gel Fig. S6 Fig. S6r

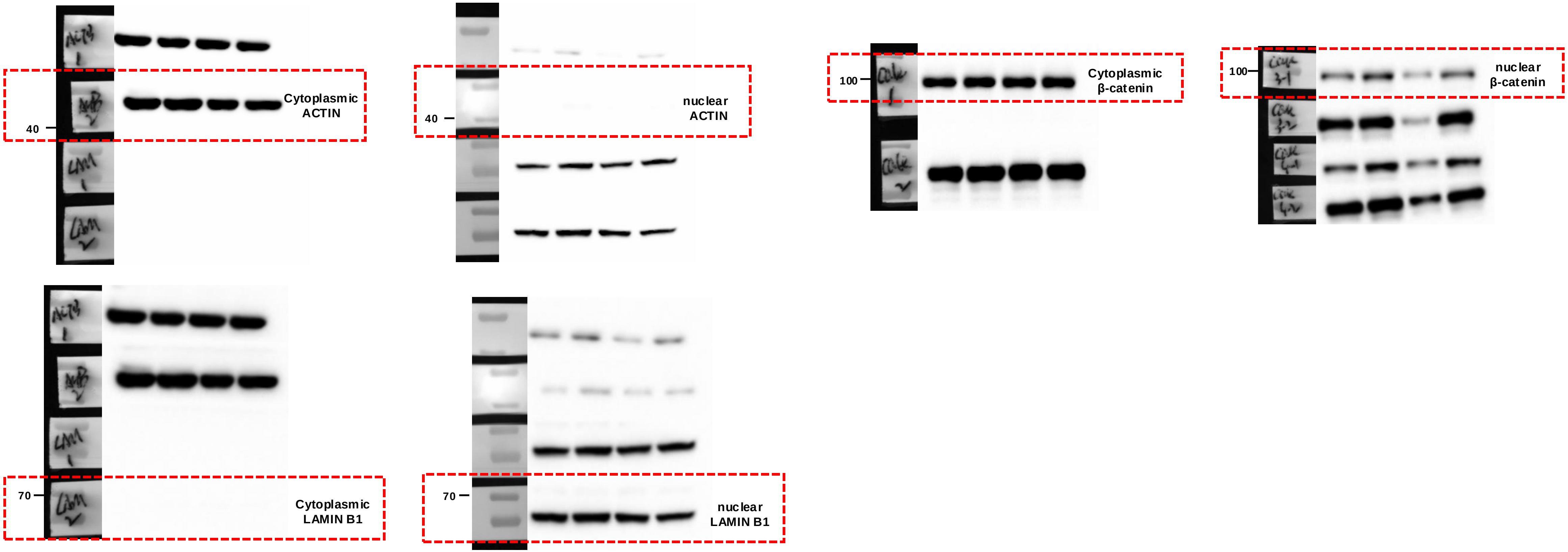

Full unedited gel for Fig. S7

Fig. S7f

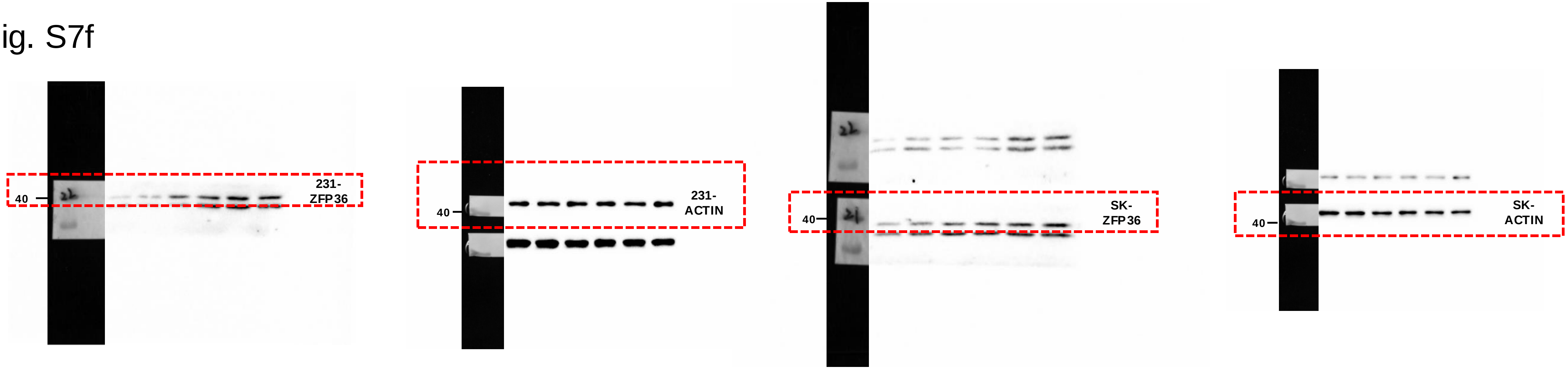

Full unedited gel for Fig. S7 Fig. S7h

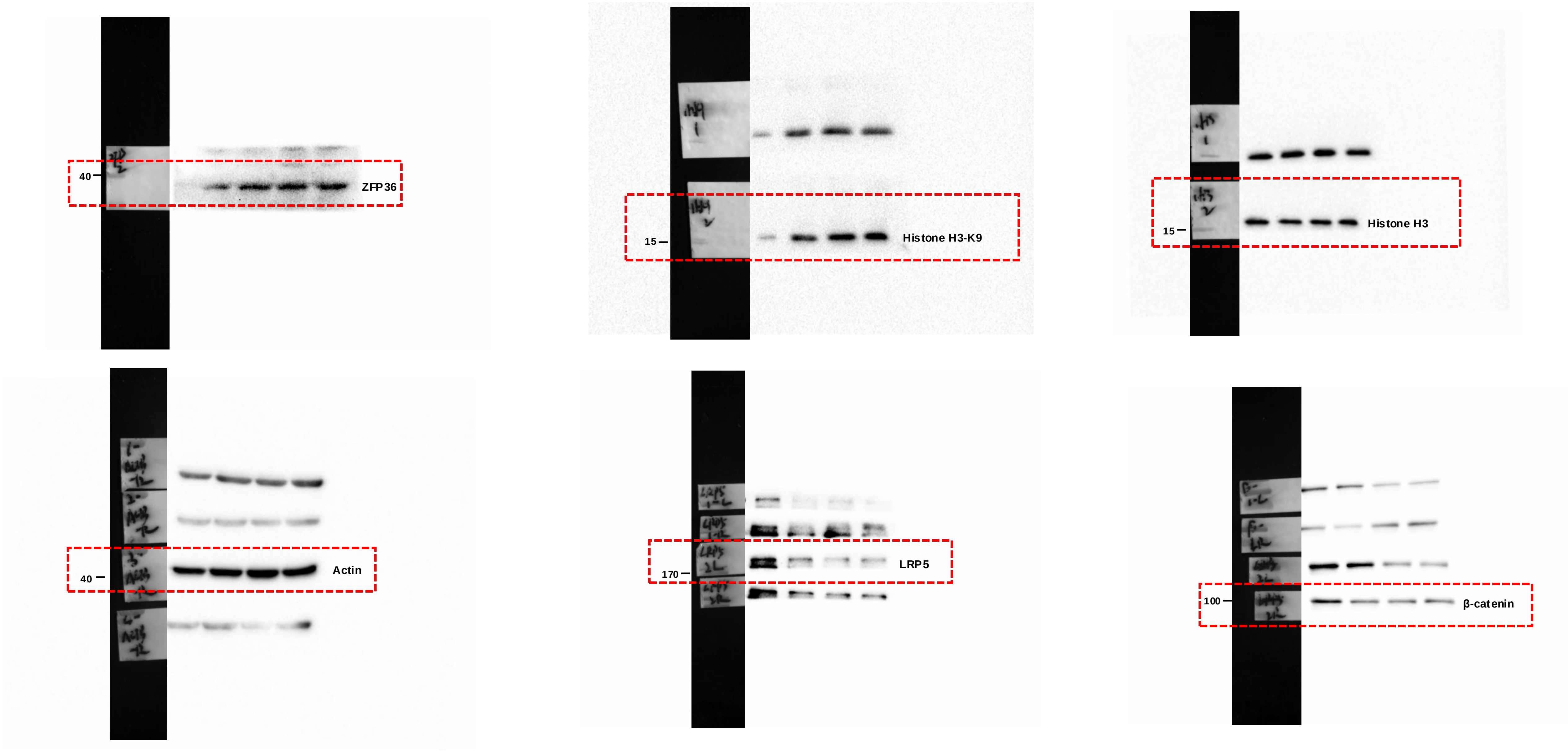

Full unedited gel for Fig. S7 Fig. S7k

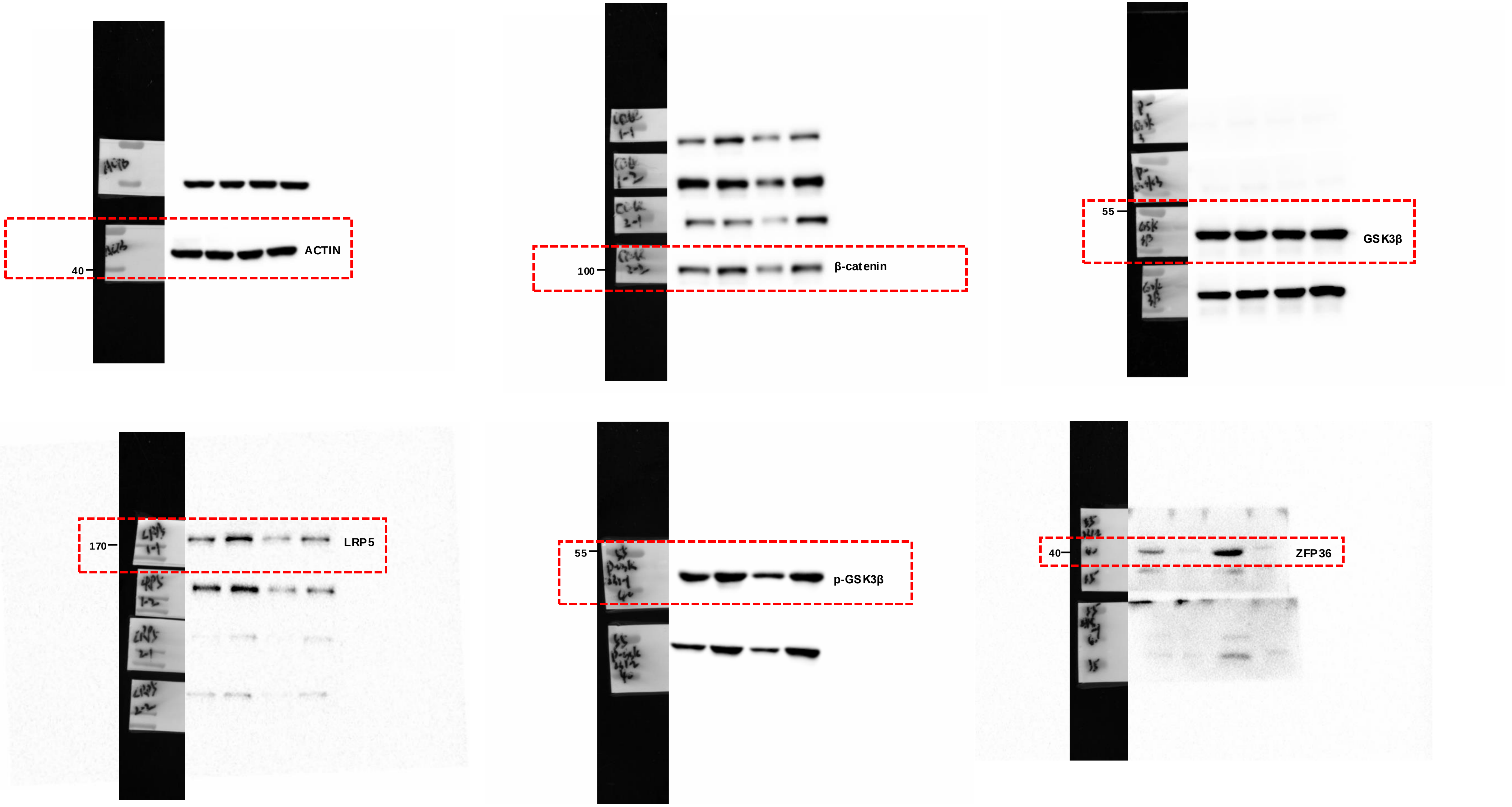

Supplement: Supplementary file 3 — Dataset2 [file 41392_2025_2159_MOESM3_ESM.pdf]
